# Intermittent Stem Cell Cycling Balances Self-Renewal and Senescence of the *C*. *elegans* Germ Line

**DOI:** 10.1371/journal.pgen.1005985

**Published:** 2016-04-14

**Authors:** Amanda Cinquin, Michael Chiang, Adrian Paz, Sam Hallman, Oliver Yuan, Indre Vysniauskaite, Charless C. Fowlkes, Olivier Cinquin

**Affiliations:** 1 Department of Developmental & Cell Biology, University of California, Irvine, Irvine, California, United States of America; 2 Center for Complex Biological Systems, University of California, Irvine, Irvine, California, United States of America; 3 Department of Computer Science, University of California, Irvine, Irvine, California, United States of America; Stanford University Medical Center, UNITED STATES

## Abstract

Self-renewing organs often experience a decline in function in the course of aging. It is unclear whether chronological age or external factors control this decline, or whether it is driven by stem cell self-renewal—for example, because cycling cells exhaust their replicative capacity and become senescent. Here we assay the relationship between stem cell cycling and senescence in the *Caenorhabditis elegans* reproductive system, defining this senescence as the progressive decline in “reproductive capacity,” i.e. in the number of progeny that can be produced until cessation of reproduction. We show that stem cell cycling diminishes remaining reproductive capacity, at least in part through the DNA damage response. Paradoxically, gonads kept under conditions that preclude reproduction keep cycling and producing cells that undergo apoptosis or are laid as unfertilized gametes, thus squandering reproductive capacity. We show that continued activity is in fact beneficial inasmuch as gonads that are active when reproduction is initiated have more sustained early progeny production. Intriguingly, continued cycling is intermittent—gonads switch between active and dormant states—and in all likelihood stochastic. Other organs face tradeoffs whereby stem cell cycling has the beneficial effect of providing freshly-differentiated cells and the detrimental effect of increasing the likelihood of cancer or senescence; stochastic stem cell cycling may allow for a subset of cells to preserve proliferative potential in old age, which may implement a strategy to deal with uncertainty as to the total amount of proliferation to be undergone over an organism’s lifespan.

## Author Summary

Stem cell cycling is expected to be beneficial because it helps delay aging, by ensuring organ self-renewal. Yet stem cell cycling is best used sparingly: cycling likely causes mutation accumulation—increasing the likelihood of cancer—and may eventually cause stem cells to senesce and thus stop contributing to organ self renewal. It is unknown how self-renewing organs make tradeoffs between benefits and drawbacks of stem cell cycling. Here we use the *C*. *elegans* reproductive system as a model organ. We characterize benefits and drawbacks of stem cell cycling—which are keeping worms primed for reproduction, and reducing the number of future progeny worms may bear, respectively. We show that, under specific conditions of reproductive inactivity, stem cells switch back and forth between active and dormant states; the timing of these switches, whose genetic control we start delineating, appears random. This randomness may help explain why populations of aging, reproductively-inactive worms experience an increase in the variability of their reproductive capacity. Stochastic stem cell cycling may underlie tradeoffs between self-renewal and senescence in other organs.

## Introduction

An important goal of aging research is not just to extend lifespan—which in *C*. *elegans* can be simply achieved by a pause in developmental and reproductive activities in the “dauer” state [1]—but to do so in a way that increases “healthspan” without diminishing organ activity. To this end, it is critical to understand whether aging is driven by organ activity or whether it is a simple function of chronological age [2]. The *C*. *elegans* gonad provides a powerful model system to address this question. Previous studies have identified mechanisms by which the “reproductive lifespan”—the period of adulthood over which *C*. *elegans* hermaphrodites can bear progeny—can be extended (e.g. [3]). But this extension does not increase the brood size, which is in fact substantially reduced. This suggests a tradeoff between reproductive lifespan and brood size, compatible with reproductive senescence being driven by reproductive activity (see also [4]). That reproductive senescence is driven by reproductive activity is however contradicted by a report that aging individuals lose “reproductive capacity”—the maximum brood size an individual is capable of producing from a given point in time until cessation of reproduction—as a function of chronological age rather than reproductive activity [2]. Here we resolve this apparent contradiction by showing that the loss in reproductive capacity—a phenomenon we refer to as “reproductive senescence” because it mimics the loss of function in other self-renewing organs—is driven chiefly not by increasing chronological age, but by activity of the gonad, and in particular by germline stem cell cycling.

To ask whether reproductive senescence is a simple function of chronological age, or whether it is driven by reproductive activity itself, it is useful to manipulate that reproductive activity (e.g. [2]). There are two naturally-occurring *C*. *elegans* sexes: males and hermaphrodites. Hermaphrodites can either self-fertilize (abbreviated as “self” below) with the ~300 stored self-sperm they produce during development, or be cross-fertilized with male sperm transferred during mating, which allows brood sizes of up to 1,200 [5]. Brood size of mated hermaphrodites is limited by senescence of the reproductive system, which ultimately stops producing fertilizable oocytes [3]. Reproductive activity can be modulated in a physiological way by controlled mating of hermaphrodites that are feminized—i.e. turned into “females”—by mutation of genes such as *fog-1* or *fog-2* [6–8]. These females do not produce self-sperm, but form an otherwise fully-developed reproductive system in which oocyte maturation and growth by cytoplasmic streaming are substantially reduced [9,10]. Females can bear progeny only after mating with males, whose sperm trigger oocyte maturation and fertilization. Virgin *fog-2* females were shown to undergo reproductive senescence at roughly the same rate as reproductively-active hermaphrodites [2] despite the fact that the ovulation rate of *fog-2* females is much reduced [9], suggesting a time-intrinsic senescence mechanism. But the mitotic zone of the germ line in feminized worms was subsequently shown to possess M-phase cells [11], suggesting that stem cells keep actively cycling even in virgin females. Reproductive senescence could therefore be driven by activity rather than being a function of chronological age.

Here, we use genetic, environmental, and pharmacological manipulations to assay the relationship between gonad activity and senescence. We establish a causal relationship between the two. We further characterize germ cell cycle behavior on a gonad by gonad basis, using a new technique we developed, and find intermittent activity. Our results strongly suggest that switching between active and inactive states is stochastic.

## Results

### More active gonads experience faster reproductive senescence

**Fig 1:**
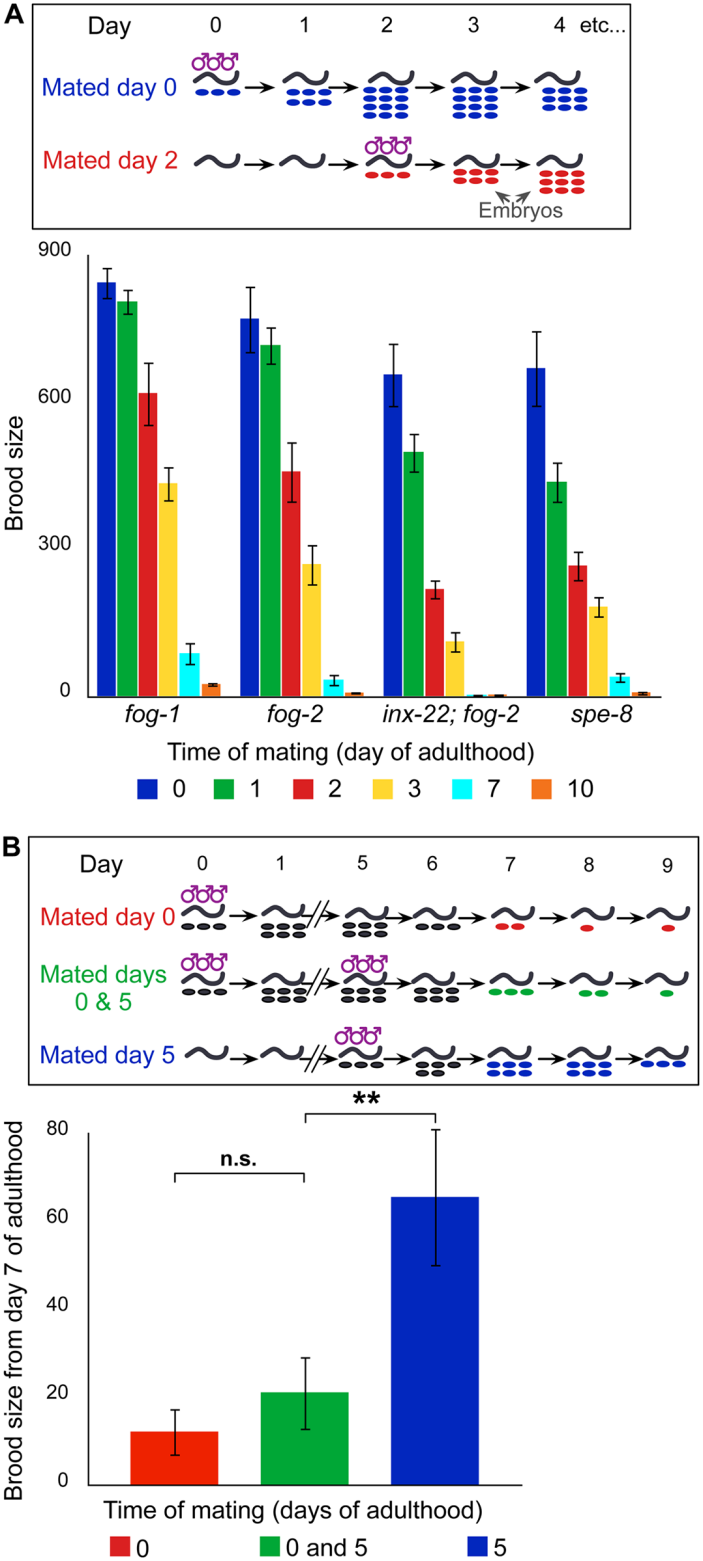
Reproductive senescence rates correlate with gonad activity. (A) Schematic of reproductive senescence assay, and brood sizes of females of various genotypes mated at increasing ages (n = 15–40 mothers for each genotype and time point). (B) Schematic of late reproductive activity assay, and brood sizes from day 7 for females mated at the onset of adulthood (day 0), on day 5 of adulthood, or both. For statistical tests see S1C Table. Error bars represent 83% confidence intervals; asterisks indicate significance of Wilcoxon rank sum test p-value.

To begin identifying causes of reproductive senescence, we followed a two-fold approach. First, we characterized loss of reproductive capacity over time in various genetic backgrounds known to differ in proximal gonad activity. Second, we asked whether high gonad activity early in life diminishes remaining reproductive capacity. To modulate proximal gonad activity, we selected *fog-1* and *fog-2* females described above, which have low ovulation rates [9,10]. We used for comparison *inx-22; fog-2* females and *spe-8* hermaphrodites—both of which are also sterile (unless mated); loss of *inx-22* results in precocious oocyte maturation in feminized gonads even in the absence of the sperm signal [9,10,12], while loss of *spe-8* preserves stimulation of oocyte maturation by self-sperm that are incapable of fertilizing the oocytes [13,14]. Virgin *spe-8* and *inx-22; fog-2* females ovulate at a rate close to wild-type (1.7/h, 0.9/h, and 2.2/h, respectively [9,14]), substantially higher than that for *fog-2* (0.2/h; n = 31), which is in turn significantly higher than that for *fog-1* (0.1/h; n = 35; p < 0.04). We mated virgins at either day 0, 1, 2, 3, 7 or 10 of adulthood and assayed total brood size (Fig 1A). We found that *spe-8* and *inx-22; fog-2* undergo faster reproductive senescence than either *fog-1* or *fog-2*. A two-way analysis of variance considering age of mating and genotype identified a significant main effect of genotype on reproductive capacity, as well as a significant interaction effect (S1A Table). For example, at day 2 of adulthood (S1 Fig) post-hoc analysis showed all pairwise differences to be significant except for the *inx-22; fog-2* / *spe-8* pair (S1B Table). Thus, across the genotypes that we studied, the ranking of reproductive senescence rates from fastest to slowest is: *inx-22; fog-2* = *spe-8* > *fog-2* > *fog-1*. Therefore, gonad activity as measured by oocyte production correlates positively with the rate of reproductive senescence.

To ascertain whether early gonad activity diminishes remaining reproductive capacity, we compared the reproductive capacities of three groups of *fog-2* females at day 7 of adulthood (Fig 1B). The first group was mated at the onset of adulthood, which causes more active germ cell cycling (see below for detailed cell cycle analysis). To verify that females in the first group did not run out of sperm late in life, a second group was mated both at the onset of adulthood and again at day 5, which did not lead to a significant increase in brood size (S1C Table; see also [2]). The third group was only mated at day 5 and had more than 3 times as many progeny after mating as the first or second group did over the same period (S1C Table). Therefore, consistent with [4], increased germline activity caused by mating is associated with hastened reproductive senescence.

### Arresting germline activity by starvation delays reproductive senescence

**Fig 2:**
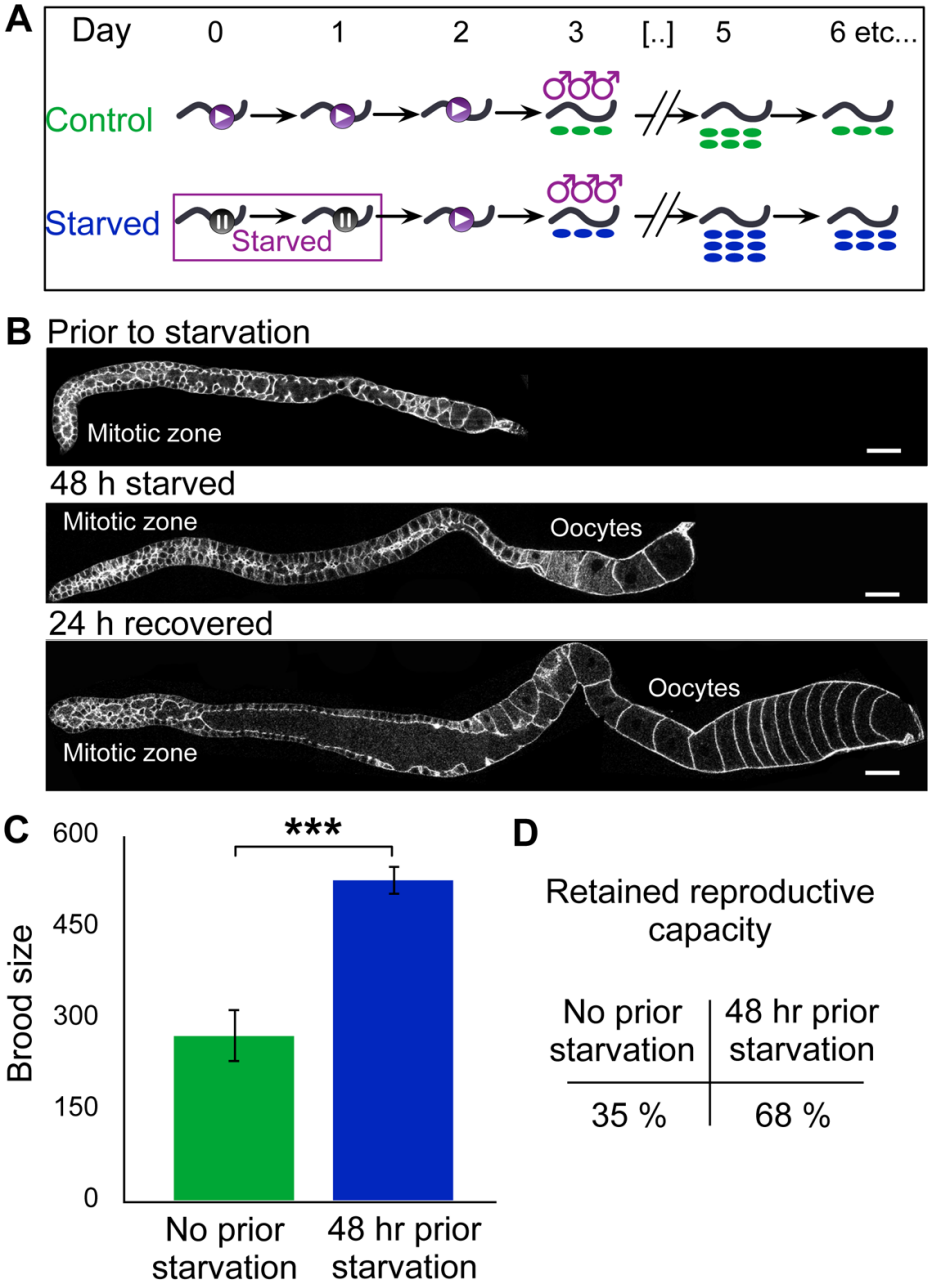
Starvation delays reproductive senescence. (A) Schematic of starvation experiment. (B) Phalloidin-stained fog-2 gonads before and after starvation. Scale bar: 25 μm. (C) Total brood size for *fog-2* females mated after 2 days of starvation or control treatment. For statistical tests see S2C Table. (D) Reproductive capacity retained by *fog-2* females after 2-day starvation or control treatment (computed by dividing numbers used in C by reproductive capacity at day 0). Error bars represent 83% confidence intervals; asterisks indicate significance of Wilcoxon rank sum test p-value.

We next asked whether strong inhibition of germline activity using conditions likely to be encountered in the wild also led to a delay in reproductive senescence. We starved *fog-2* females from the last larval stage (L4) for two days (Fig 2A). Despite being starved and experiencing a ~30-fold drop in germline mitotic index (S2A Table), females progressed from L4 to the adult stage and produced fully-formed oocytes. We did not observe shrinking of the germ line or the reproductive diapause identified in wild-type by [15] (Fig 2B), likely because the absence of sperm in females prevents the redirection of resources to slowly-growing embryos proposed by [16]; consistent with this, starvation for 2 days markedly reduced the number of apoptotic cells identified in females using a feminized *ced-1*::*gfp* reporter strain, from 18.3 per gonadal arm to just 1.9 (S2B Table and S2 Fig; note that this is in contrast to starvation of hermaphrodites over a 6-hour period [17]). We then returned the females to food for 24 h, mated them with males, and assayed their brood sizes. Compared to continuously-fed controls of the same age, brood size was increased by almost 2-fold (S2C Table and Fig 2C). This increase occurred even as apoptosis is restored to normal levels when females are returned to food (S2D Table and S2 Fig). Starved worms thus retain a higher percentage of their reproductive capacity during the starvation period compared to controls (Fig 2D). While starvation has pleiotropic effects such as increased stress resistance [18], these results are also compatible with the idea that germline activity—measured either by germ cell cycling or oocyte production—drives reproductive senescence.

### Germ cell cycling drives reproductive senescence

**Fig 3:**
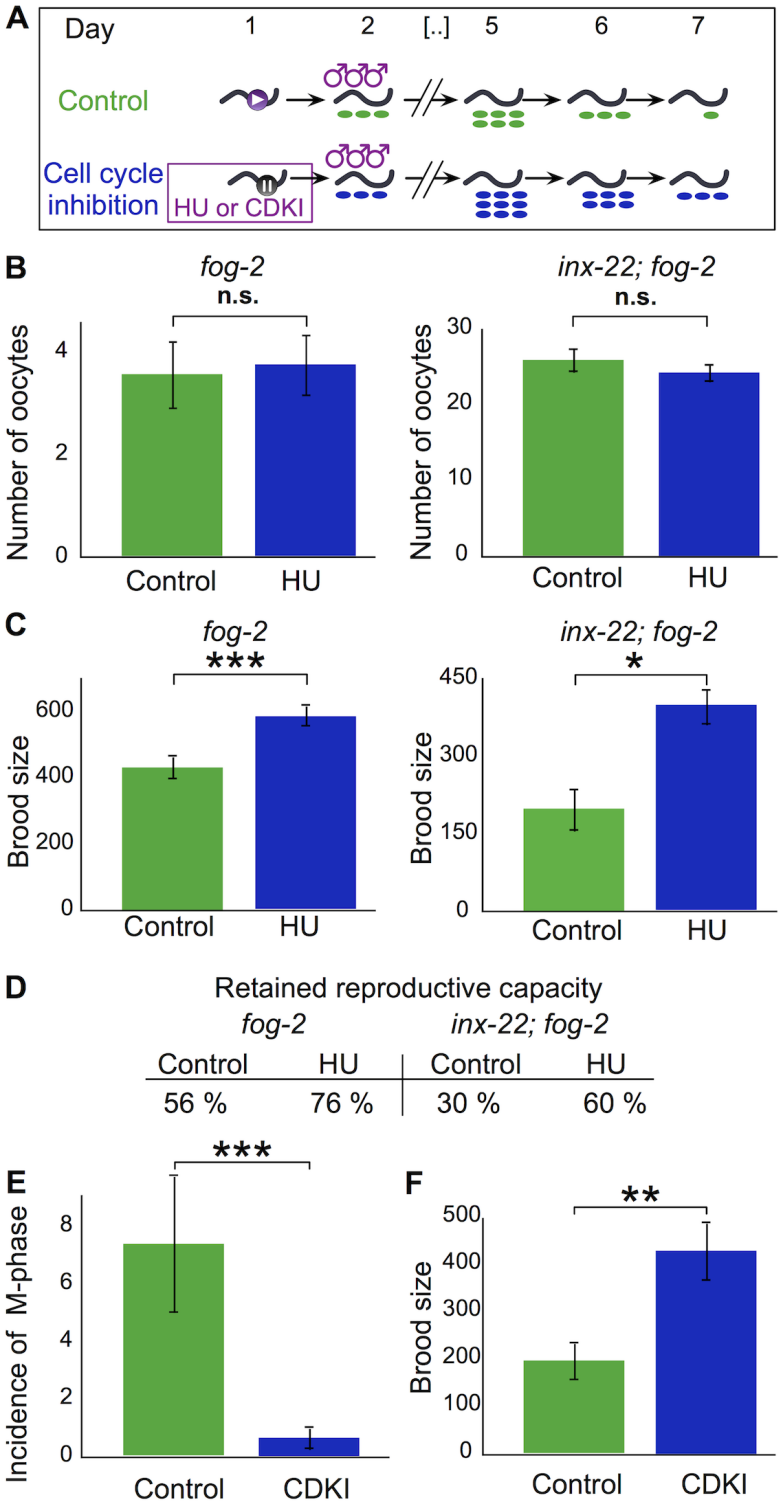
Stem cell cycling drives reproductive senescence. (A) Schematic of cell cycle inhibitor experiments. (B) Total number of oocytes produced by fog-2 or inx-22; fog-2 females during the HU treatment window. For statistical tests see S3A Table. (C) Brood size for fog-2 or inx-22; fog-2 females mated after the HU treatment window. For statistical tests see S3B Table. (D) Reproductive capacity retained by fog-2 or inx-22; fog-2 females after HU or control treatment (computed by dividing numbers used in C by reproductive capacity at day 0). (E) Incidence of cells in M-phase following 24 h treatment with CDK inhibitor Roscovitine or control treatment with DMSO only. For statistical tests see S3D Table. (F) Brood size for fog-2 females mated after the Roscovitine treatment window. For statistical tests see S3E Table. Error bars represent 83% confidence intervals; asterisks indicate significance of Wilcoxon rank sum test p-value.

To ask directly if reproductive senescence is driven by germline activity, and to distinguish between the influence of cell cycling distally and oocyte maturation proximally, we next performed cell cycle inhibition experiments (Fig 3A). We fed the small molecule hydroxyurea (HU), a specific inhibitor of DNA synthesis (germ cells are the only mitotically active cells in adults). We found that HU treatment substantially reduces the incidence of M-phase cells within 12 h (from 3.2 to 0.35; n = 18 and 17 respectively) and eliminates it after 1 day (n = 20). We tested whether germ cell cycle arrest was accompanied by a reduction in ovulation rate by exposing virgin females to HU for 24 h at day 1 of adulthood and counting the number of oocytes laid during this period. We found that for both *fog-2*, which has a low ovulation rate, and *inx-22; fog-2*, which has a much higher ovulation rate, there was no effect of HU treatment on ovulation (S3A Table and Fig 3B). We compared reproductive capacities of females that prior to mating received a 24 h treatment with HU or a control treatment without HU. HU treatment increased *fog-2* reproductive capacity by 27% (S3B Table and Fig 3C) and increased *inx-22; fog-2* reproductive capacity by 50% (S3B Table and Fig 3C). Cell-cycle arrested females thus retain a higher percentage of their reproductive capacity compared to controls (Fig 3D). HU treatment has a number of effects other than cell cycle arrest. While these effects are detrimental [19,20] and are expected if anything to hasten senescence, the possibility remained that HU had a hormetic effect. To test this idea we asked whether HU treatment increased lifespan or thermotolerance, but found that it in fact decreased them (S3C Table and S3A and S3B Fig). While this does not formally exclude the possibility of a hormetic effect on *reproductive* lifespan, it shows that HU does not have a global beneficial effect on worm health. Overall, the increase in brood size that results from the HU treatment is thus remarkable.

To confirm that delayed reproductive senescence induced by HU is not an off-target effect, we also performed a cell cycle inhibition experiment using the selective cyclin-dependent kinase inhibitor (CDKI) Roscovitine. Roscovitine has previously been used to reversibly inhibit mitosis in starfish and sea urchin embryos [21]; we found that it effectively reduces the mitotic index in worm germ lines (S3D Table and Fig 3E). We found that the reproductive capacity of females treated with Roscovitine for 24 h prior to mating was over 2-fold larger than that of females that received a DMSO control treatment (S3E Table and Fig 3F). While HU- and Roscovitine-treatment brood sizes cannot be compared directly, because treatment with DMSO—used as a Roscovitine solvent—decreases brood size [22], the qualitative effects of HU and Roscovitine treatments are the same.

### DNA damage response increases with cycling and curtails reproductive capacity

The role that we uncovered for cell cycling in driving reproductive senescence, combined with the role of DNA damage in driving senescence in other systems [23], led us to wonder if cell cycling could curtail reproductive output at least in part through the DNA damage response (DDR).

**Fig 4:**
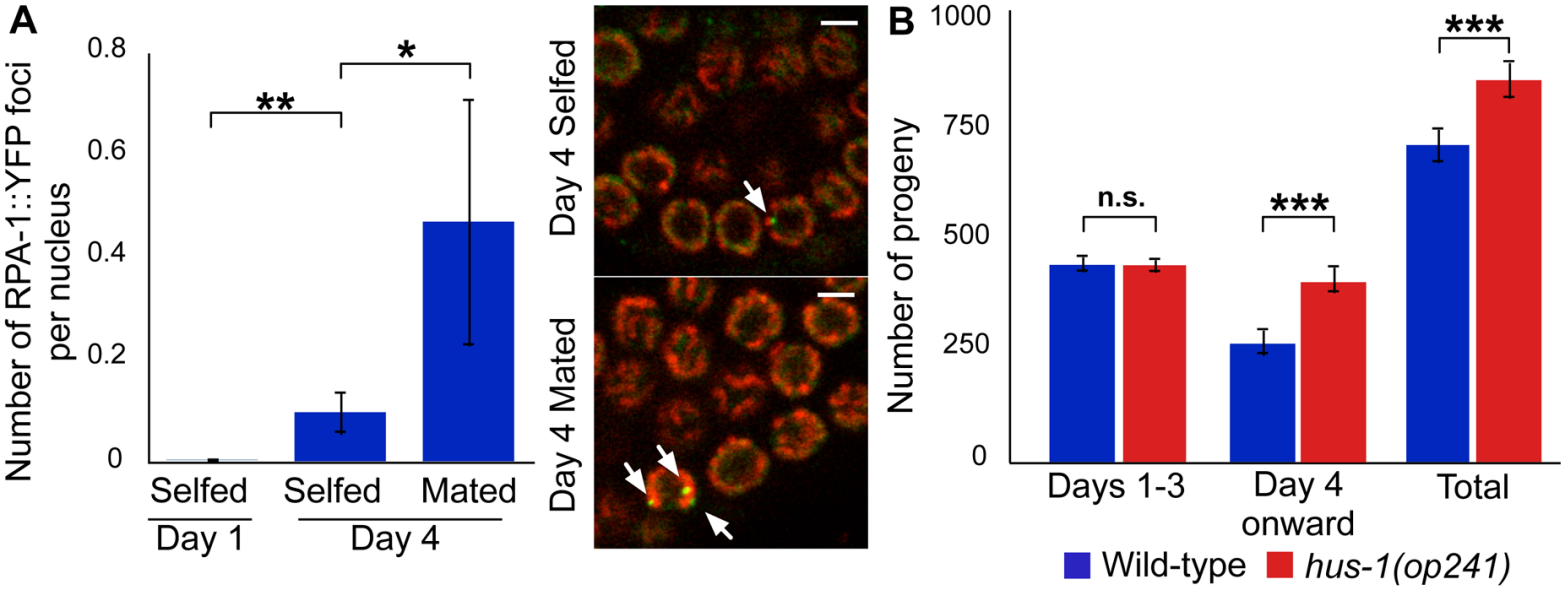
DDR increases with past reproductive activity and curtails reproductive capacity. (A) Average number of RPA-1::YFP foci per nucleus in late pachytene. Image panels show example RPA-1 foci (arrows; YFP: green; DNA: red). Scale bars: 2.5 μm. For statistical tests see S4A Table. (B) Total brood size is larger for mated hus-1(op241) (red bar) than for mated wild-type (blue bar). For statistical tests see S4B Table. Error bars represent 83% confidence intervals; asterisks indicate significance of Wilcoxon rank sum test p‑value. To test whether the DDR curtails reproductive activity, we utilized the *hus-1* reduction of function allele *op241*. This mutation abrogates multiple forms of DDR [30] but preserves normal reproductive activity in selfed worms [31]. Mated *hus-1(op241)* hermaphrodites had a significantly larger brood size than wild-type controls (S4B Table and Fig 4B); the reproductive schedule was almost identical between days 1 and 3, but from day 4 onward *op241* had a ~1.5-fold greater number of progeny. This shows that the DDR hastens reproductive senescence, by a mechanism that remains to be identified.

To test whether cell cycling leads to increased DDR, we focused on the single stranded DNA binding Replication Protein A (RPA-1 in worms). While RPA-1 is activated in response to multiple forms of DNA damage [24–26], it plays in particular a role in the repair of induced double strand breaks (DSBs) associated with meiotic crossovers. This role leads to presence of RPA-1 foci in the pachytene stage of meiosis [27,28]. Defects in chromosome segregation in the mitotic zone do not lead to increased prevalence of foci in the mitotic zone itself, but instead lead to accumulation in late pachytene of meiosis-induced DSBs that are not resolved [29]. We quantified RPA-1::YFP foci in pachytene (zones 5 and 6 defined by [28]), in young *rpa-1*::*yfp* hermaphrodites taken at day 1 of adulthood, and in *rpa-1*::*yfp* hermaphrodites taken at day 4 of adulthood that had been selfed or that had been mated at day 0 of adulthood. We found over ~5-fold more foci per nucleus in the mated group than in either of the selfed groups (S4A Table and Fig 4A). Consistent with the pattern of increased cycling being associated with increased RPA-1 foci, selfed hermaphrodites had more foci at day 4 than at day 1 (S4A Table). Overall, although it remains to be established how increased cycling may lead to chromosome segregation defects or other defects in the mitotic zone, these results strongly suggest that this increased germ cell cycling leads to increased proximal germline DDR.

### Dose-dependent relationship exists between cell cycling and progression of reproductive senescence

Our results show that gonad activity strongly contributes to reproductive senescence, mostly as a result of germ cell cycling. We thus hypothesized that, at any given point in the reproductive life of a worm, remaining reproductive capacity is inversely related to the total amount of germ cell cycling that has occurred up to that point. To test this hypothesis, we decided to ask whether a dose-dependent relationship exists between average cell cycle rate and the rate of reproductive senescence by using genetic or environmental mutations that modulate cell cycling. To identify suitable genetic manipulations, we compared *fog-1* and *fog-2* females with *inx-22; fog-2* and *spe-8*. Since the former two strains undergo slower reproductive senescence than the latter (see above; Fig 1A) and have lower proximal gonad activity, we surmised that they might also have slower distal cycling. We also assayed for changes in cell cycle when oogenesis rates are high due to the presence of self or male sperm, or low due to depletion of self sperm. We first detail our cell cycle analysis, and subsequently compare results to reproductive senescence data.

**Fig 5:**
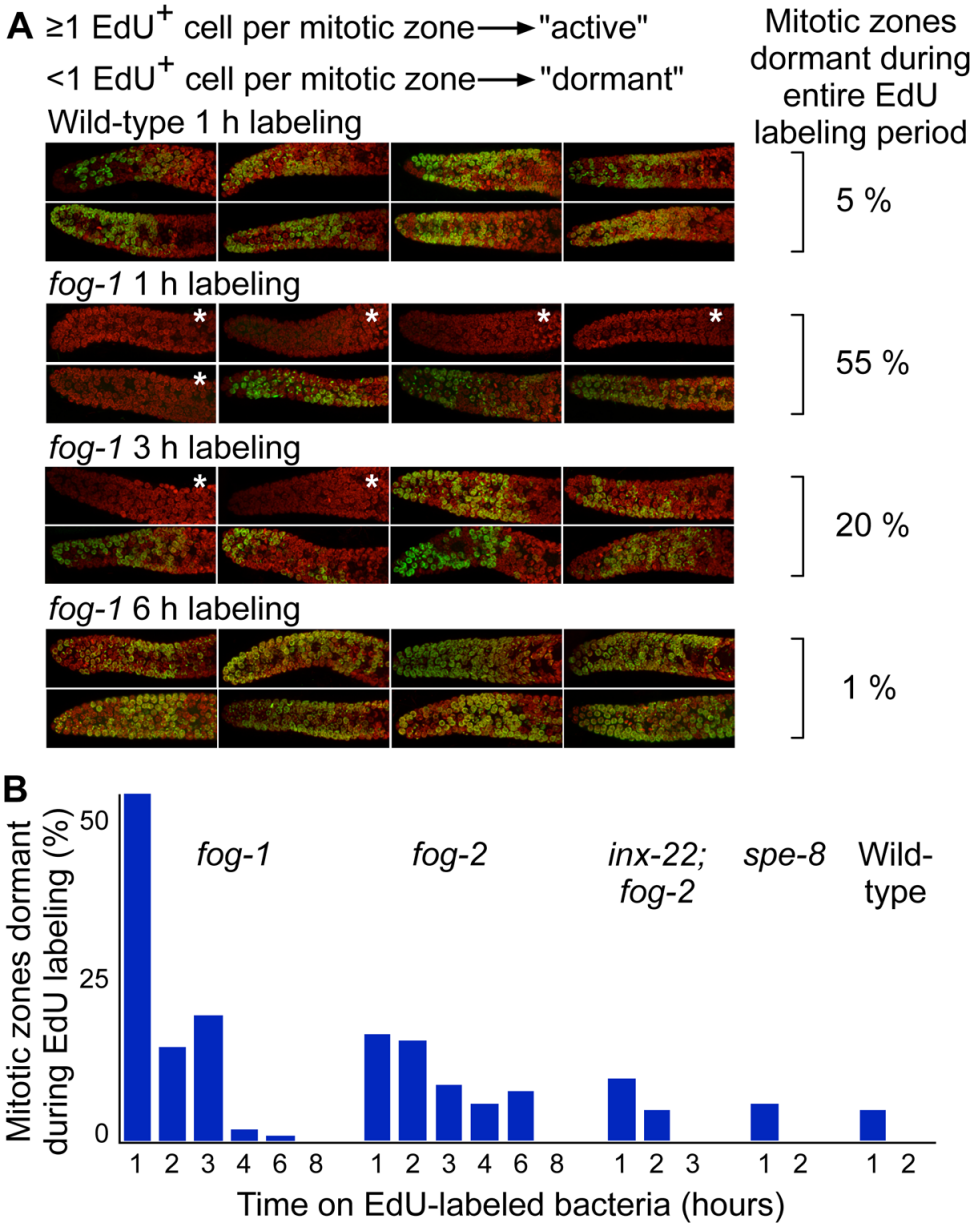
Mitotic zones in gonads with reduced reproductive activity intermittently occupy a dormant state. (A) Representative z-projection images of continuous EdU labeling of wild-type and fog-1 (DNA: red; EdU: green). “Dormant” mitotic zones are defined by the absence of any EdU positive cell, and are marked with an asterisk. Almost all wild-type mitotic zones show activity within 1 h of continuous labeling, whereas fog-1 mitotic zones take in excess of 6 h to all have experienced activity. (B) Fractions of day 1 mitotic zones remaining unlabeled as a function of time on EdU-labeled food (n = 40–82 for each genotype and time point).

We first attempted to determine average cell cycle speeds. To this end we carried out pulse-chase experiments using bacterial food labeled with 5-Ethynyl-2’-deoxyuridine (EdU), which is incorporated by cells in S phase. Although virtually all young, selfed wild-type mitotic zones contained EdU-positive cells following a 30-minute pulse (consistent with previous reports; [32,33]), many mitotic zones from virgin females contained no labeled cells. If this lack of S-phase labeling was due to all cells in a given gonad being found by chance in G1-, G2-, or M-phase, given that M-phase length is about 10% that of G1, G2, and M combined (e.g. [33,34]), and mitotic zones contain ~260 cells (S5E Table), one would expect to find on average in such mitotic zones 10% of 260 cells, i.e. 26 cells, in M-phase. Since neither we nor others [35] have observed mitotic zones with such a large number of M-phase cells, lack of any S-phase cell in a mitotic zone indicates that the mitotic zone as a whole is in a dormant state. The two most likely explanations for the presence of unlabeled mitotic zones were thus either that the unlabeled worms had not ingested the EdU-labeled bacteria, or that the mitotic zones were dormant at the time of the pulse. To explore these possibilities, we switched to a continuous EdU labeling assay. In wild-type gonads at day 1 of adulthood, virtually all mitotic zones had at least one labeled cell within the first time point we assayed, with only 5% remaining unlabeled (1 h labeling; see example images in Fig 5A and detailed graphs in Fig 5B). The proportion of unlabeled mitotic zones at 1 h was substantially higher for *fog-1* (55%) and for *fog-2* (17%) than for wild-type; it took in excess of 6 h for labeled cells to have appeared in all feminized mitotic zones (Fig 5A and 5B). To test whether labeling delays could be due to pauses in feeding, we fed virgin worms bacterial food mixed with fluorescent beads. At the very first time point we assayed (1 h), 100% of wild-type, *fog-1*, and *fog-2* worms had ingested the fluorescent beads (see example in S4A Fig). This strongly suggests that different labeling rates are due to *bona fide* differences in mitotic zone cycling, with a substantial proportion of *fog-1* and *fog-2* gonads being in a dormant state in which germ cells are not progressing through the cell cycle.

Continuous EdU labeling results show the existence of at least two states in which reproductively-inactive gonads can reside—an actively-cycling state, and a dormant state—and suggest that stochastic switching occurs between these states. Specifically, that different cycling states exist within the population of feminized, reproductively-inactive gonads can be inferred from the large differences in labeling times for e.g. *fog-2* gonads (the same reasoning applies to *fog-1* gonads): some gonads label almost immediately, showing that they are actively progressing through S-phase at the time of label application, while others take up to 8 h and thus do not have cells actively progressing through S-phase during these 8 h. If *fog-1* and *fog-2* simply cycled continuously, in the same way as wild-type but with uniform slowing down of all cell cycle phases, the distribution of cell cycle phase indices would be unchanged and the number of gonads with at least one cell progressing through S-phase would be the same as in wild-type at any given time—and the continuous labeling time courses would therefore appear identical for all genotypes. Stochasticity can be inferred from the fact that EdU labeling of reproductively-inactive gonads happens immediately for some gonads but takes up to 8 h for others: this shows independent behavior of gonads with identical genotypes, for which stochastic switching between active and dormant states is the most parsimonious explanation. To begin confirming the existence of such states by independent means, we quantified the coefficient of variation (CV) in mitotic index. The CV is defined as the ratio of standard deviation to mean and thus provides a unit-free measure of noise. The *fog-1* and *fog-2* CVs were 2.3- and 1.7-fold higher, respectively, than the wild-type CV, differences that are significant at a 95% confidence level (S5A and S5B Table). These differences show a larger spread among the population of the mitotic index measured at a given point in time, as expected if there is a mixture of gonads with little or no mitosis (the dormant subpopulation) and gonads cycling as normal (the active subpopulation). This supports the idea that feminized gonads switch back and forth between active and dormant states. This idea is further tested by independent means below.

We asked whether the dormant state we identified was an artificial byproduct of mutations in *fog-1* or *fog-2*, or whether it was a state naturally occupied by sperm-deprived gonads. We first performed continuous EdU labeling of selfed wild-type hermaphrodites taken at day 3 of adulthood, by which time reproductive activity has started declining; full labeling took 4 h (S4B Fig), twice as long as for hermaphrodites at day 1 (which are fully active). Mitotic zones from mated hermaphrodites assayed at day 3 of adulthood, however, labeled at the same speed as selfed worms at day 1 (S4B Fig). We next performed continuous EdU labeling using *inx-22; fog-2* or *spe-8* gonads, which do not produce embryos but retain sustained oocyte maturation. The fractions of dormant *inx-22; fog-2* and *spe-8* mitotic zones were closer to that of wild-type (Fig 5B) than to that of *fog-1* or *fog-2*. Similarly, *inx-22; fog-2* and *spe-8* times to labeling of all mitotic zones were closer to that of wild-type than to that of *fog-1* or *fog-2* (Fig 5B). The differences in median labeling times were significant for all genotype pairs (p < 7.8E-3, Wilcoxon test with Bonferroni correction for 15 tests; see Methods), except for pairs taken within the group formed by wild-type, *spe-8*, and *inx-22; fog-2*, which all behave similarly (p > 0.05). Differences in fractions of dormant gonads matched differences in mitotic index CV: the CV for *fog-1* was significantly higher than that for *fog-2*, which in turn was significantly higher than the CV for wild-type, *inx-22; fog-2* or *spe-8* (S5B Table). Finally, we asked whether the behavior we observed was particular to *C*. *elegans*, or whether it was shared with other nematode species. We chose *C*. *remanei* [36], for which genetic manipulation is unnecessary for feminization because it is a male/female species, but which undergoes reproductive senescence much like *C*. *elegans* females (S4C Fig). We found that the fraction of dormant mitotic zones was 27% for virgin *C*. *remanei* females, but that mating decreased that fraction to 5% (S4D Fig). Therefore, the mitotic zones are dormant at a substantially higher frequency in reproductively-inactive gonads than in reproductively-active gonads, both in *C*. *elegans* and in the related nematode species *C*. *remanei*.

**Fig 6:**
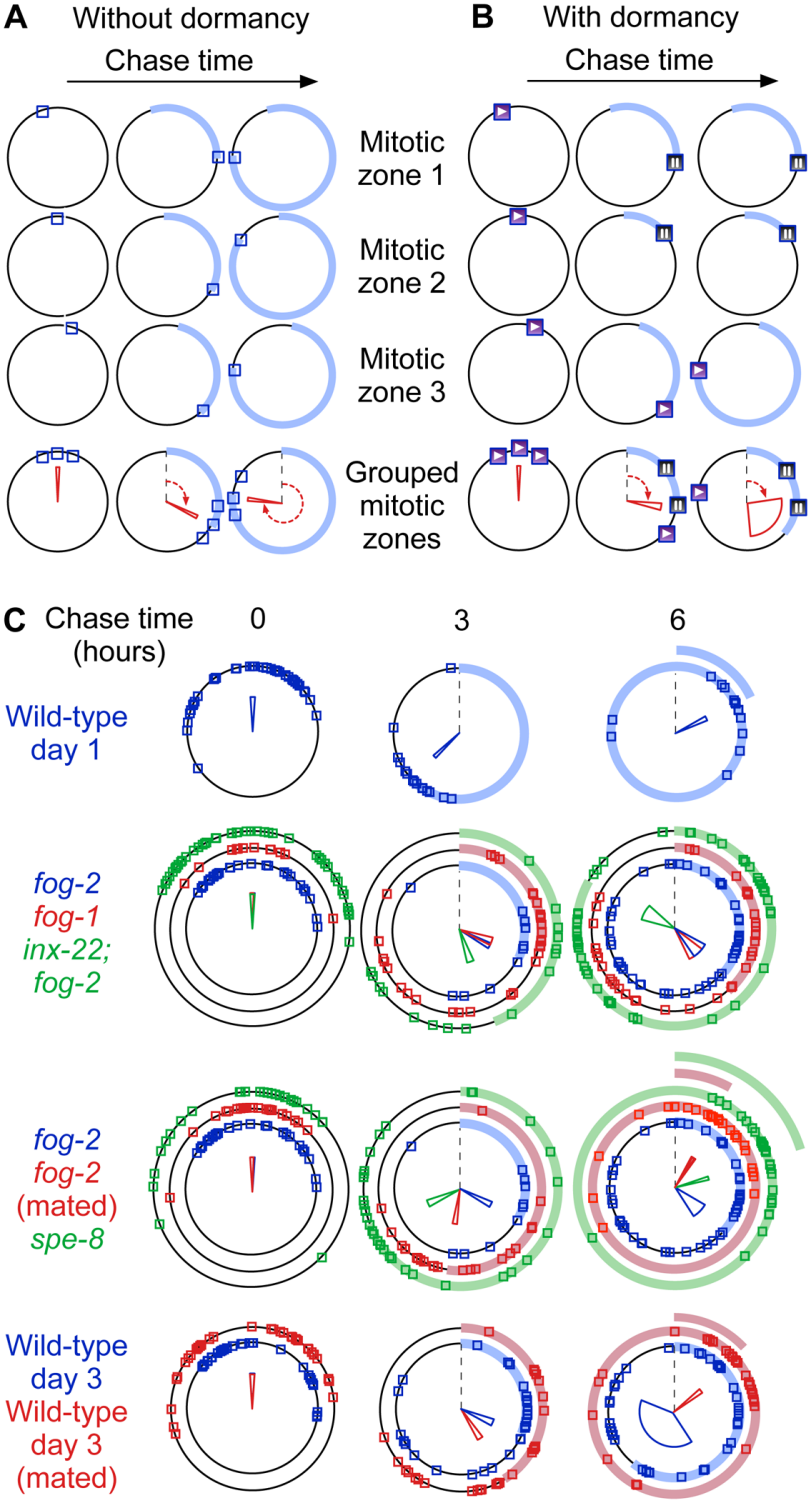
Slower average cell cycle progression and loss of synchrony in gonads with reduced reproductive activity. (A, B) Principle of EdU pulse chase analysis. Three fictitious mitotic zones cycle steadily (A) or stochastically enter a dormant state (B; play and pause symbols within the squares). The position of each square on the circle represents mitotic zone cell cycle progression (progression from time of labeling highlighted by a colored band). A full revolution on the circle corresponds to all cells in the mitotic zone having undergone a full cycle. The red wedge in the bottom row shows average cycle progression (angle shown by red arrows) and the amount of dispersion between mitotic zones (width of the wedge, set to the inverse of the magnitude of the resultant computed as the vector sum of individual gonad positions). (C) Analysis of cell cycle progression after EdU pulse-chase. Mitotic zones from virgin females or old wild-type hermaphrodites lose synchrony at later chase times, as shown e.g. by wider wedges (virgin fog-2 data is repeated in rows 2 and 3 to facilitate comparisons). See also S5 Fig, S5 Table, S1 Movie, S1 Dataset and S1 Text.

Having established that less active gonads experience periods of cell cycle dormancy, we returned to our pulse-chase dataset. To quantify cell cycle rates of active mitotic zones, and to verify that mitotic zones switch back to the dormant state from the active state, we developed a method that, instead of relying on population averages, computes cell cycle progression of individual mitotic zones. We segmented individual cells in confocal stacks of mitotic zones that were fixed and stained for DNA and EdU, and fit the data to computational simulations of germ cell cycling (see S1 Text). Overall progression of mitotic zones through the cell cycle is computed as a phase that defines a position on a circle, with one full revolution corresponding to a complete cell cycle (Fig 6A). A mitotic zone that labels during the EdU pulse but becomes dormant during the chase would stop progressing around the circle (Fig 6B). We validated our technique using wild-type young adult mitotic zones, for which a full revolution took ~5.5 h to complete (Fig 6C); this estimate of cell cycle length is consistent with previous findings [33,37]. We then compared initial cell cycle progression during the EdU chase between hermaphrodites and virgin females. Initial progression rates (i.e. rates of non-dormant mitotic zones) of wild-type, *fog-1*, *fog-2*, *inx-22; fog-2* or *spe-8* were largely similar (S5C Table). We estimated average cycling rates as the product of the fraction of active mitotic zones and their initial progression rates. Rankings are: wild type (0.17 cycles / h) ≃ *spe-8* (0.20 cycles / h) ≃ *inx-22; fog-2* (0.18 cycles / h) > *fog-2* (0.13 cycles / h) > *fog-1* (0.08 cycles / h), which are equivalent to the rankings for reproductive senescence rates. We do not know the molecular basis for the difference in average *fog-1* and *fog-2* cell cycle rates—slower oocyte maturation in *fog-1* (S4E Fig) could increase mitotic zone dormancy—but in any case this difference provides a useful tool to investigate the effects of cell cycling activity.

### Reproductively-inactive mitotic zones switch between dormant and actively-cycling states

To ask if mitotic zones switch from the active to the dormant state, similar to switching from the dormant to the active state shown by continuous EdU labeling experiments, we further assayed the cell cycle progression of mitotic zones pulsed with EdU. Individual mitotic zones of young adult hermaphrodites pulsed with EdU showed little dispersion in their overall cell cycle progression, even after 6 h of chase—covering over a full cycle length (as shown by visual inspection of phase plots and by quantification of inter-gonad synchrony depicted as wedge width in Fig 6C). This shows that reproductively-active gonads have mitotic zones that progress through the cell cycle in a highly-similar fashion. By contrast, individual mitotic zones from virgin, feminized gonads showed substantially larger dispersion along the set of possible phases as shown by increased wedge width in Fig 6C, 6 h time point, for virgin *fog-1* and virgin *fog-2* (compare to wild-type day 1 or to mated wild-type day 3). Dispersion was even stronger in wild-type hermaphrodites at day 3—to the point that cell cycle progression appeared largely randomized. Given that initial progression rates are highly similar across the genotypes and conditions that we tested, the simplest interpretation of these results is that the time at which labeled mitotic zones return to the dormant state is stochastically distributed. To test whether dispersion in the amounts of cell cycle progression after labeling was due to reduced reproductive activity, we measured cell cycle activity in mated young females, and in hermaphrodites mated at day 1 of adulthood and assayed at day 3. In both cases, the increase in reproductive activity caused by mating was accompanied by a switch of cell cycle behavior to that of young adult hermaphrodites (Fig 6C) and by a reduction in the mitotic index CV (S5B Table), showing that occupancy of the dormant state is regulated by reproductive activity. Therefore, gonads with reduced reproductive activity switch back from the actively-cycling state to the dormant state in a way that is in all likelihood stochastic.

**Fig 7:**
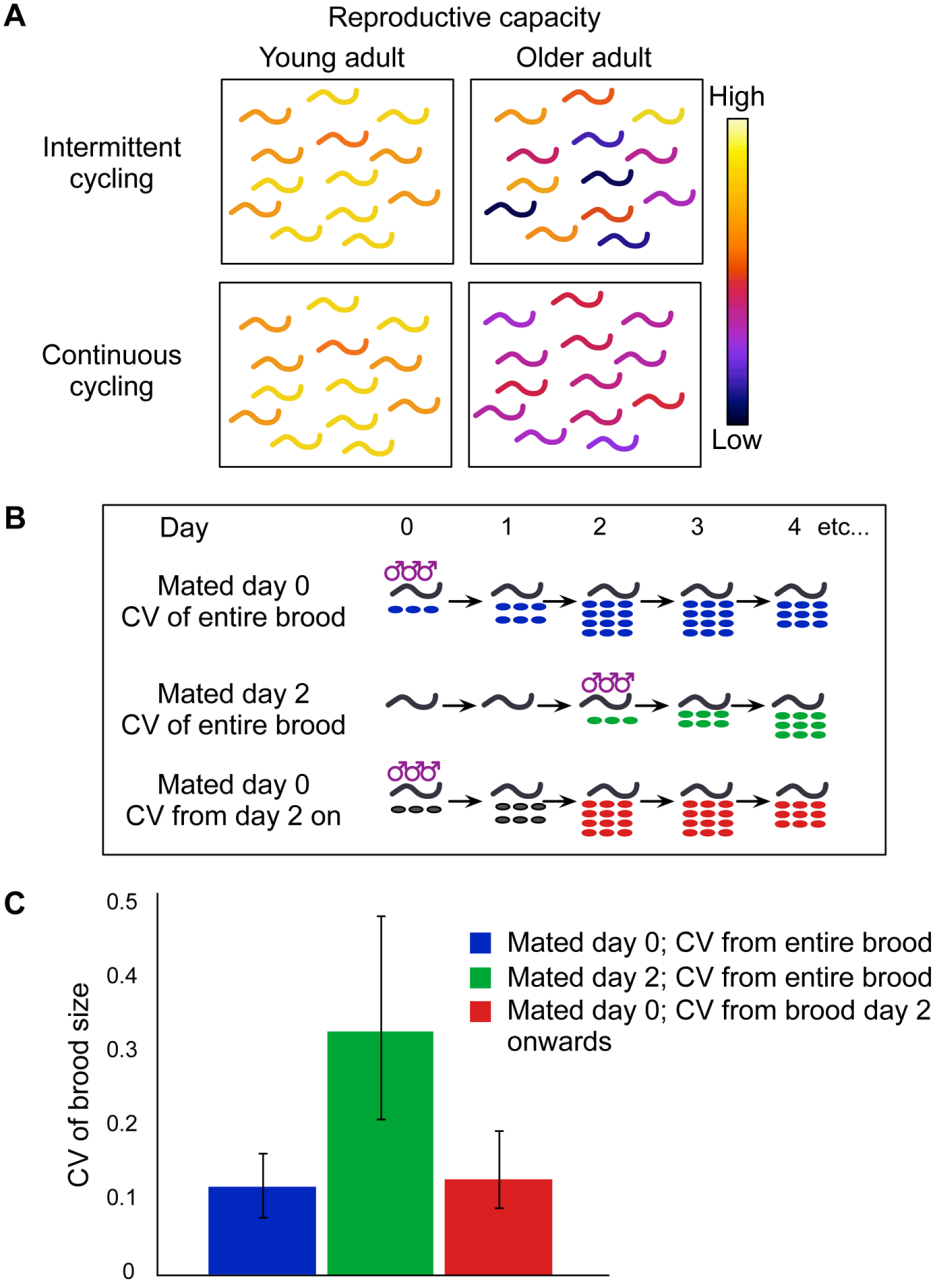
Variability of remaining reproductive capacity in aging populations. (A) According to a model by which gonads switch stochastically between dormant and active states, and according to which activity leads to progressive loss of reproductive capacity, there may be an increase with age in the population-level spread of remaining reproductive capacity. Color coding for worms is based on remaining reproductive capacity (lookup table on right). (B) Schematic of experiments used to measure reproductive capacity CV. (C) Reproductively-inactive female populations undergo an increase in reproductive capacity CV. Error bars represent 99% confidence intervals; for details see S6 Table).

A prediction from a model whereby feminized gonads switch back and forth stochastically between active and dormant states, and whereby gonad activity leads to loss of reproductive capacity, is that there may be an increase in variability of remaining reproductive capacity as the population ages (Fig 7A). This is because stochasticity of switching may lead to mitotic zones spending different total amounts of time in the active state. We thus tested whether inter-individual variability in remaining reproductive capacity does increase with age. To this end, we computed the coefficients of variation (CVs) of brood size in *fog-1* populations mated at different times (Fig 7B). The CV is defined as the ratio of standard deviation to mean and thus provides a unit-free measure of noise. When mated at the onset of adulthood (day 0), the brood size CV was 0.12 (Fig 7C and S6 Table). By contrast, the brood size CV for mating on day 2 of adulthood was 0.33, i.e. ~3 fold higher. To distinguish between a simple increase in variability associated with aging (as is observed for many phenotypes; [38]) and an increase in variability driven by stochastic cell cycling, we considered brood sizes computed from day 2 of adulthood onwards, for worms mated at the onset of adulthood. The CV was 0.13 (Fig 7C and S6 Table), virtually identical to the CV of brood sizes scored from day 0 and significantly lower than the CV for worms mated on day 2 of adulthood. Therefore, the age-dependent increase in variability of remaining reproductive capacity for reproductively-inactive females is stronger than the increase that occurs for reproductively-active worms. We verified using a simple simulation that, given a suitable probability distribution of times spent in the dormant and in the active state, a population of females losing the same reproductive capacity as *fog-1* over a period of two days can indeed experience a ~3-fold increase in the CV in remaining reproductive capacity (see S1 Text). Overall, these results support the idea that stochastic bursts of cycling drive reproductive senescence.

### Regulation of mitotic zone dormancy

To start querying organismal regulation of the dormant state, we first asked whether the state of a mitotic zone in one gonadal arm relates to that of the mitotic zone in the sister arm from the same worm. At 1 h of continuous EdU labeling, we found 92% agreement in EdU status (defined by presence or absence of at least one labeled cell; n = 36 pairs) between pairs of mitotic zones from the same worm; this substantial synchrony in gonadal arm states suggests that dormancy may be regulated at the organismal level, which could perhaps occur through neuronal control of TGF-beta signaling [39] or insulin signaling [40,41].

**Fig 8:**
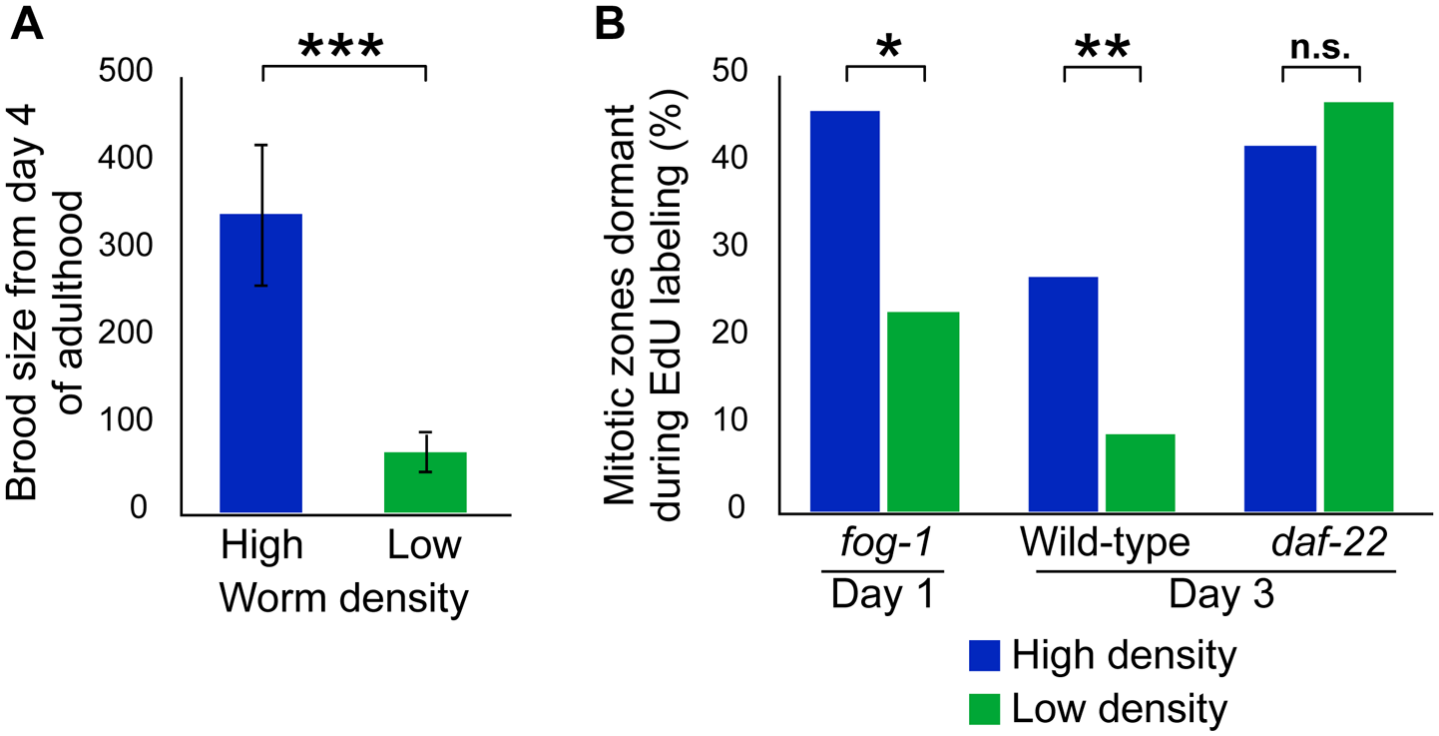
Population density influences intermittent cycling in a dauer pheromone dependent fashion. (A) Brood size of fog-1 females that had been kept at low or high density, and then mated at day 4 of adulthood (for statistical tests see S7A Table). Error bars represent 83% confidence intervals. Asterisks indicate significance of Wilcoxon rank sum test p‑value. (B) The percentage of mitotic zones that were in the dormant phase during a 1 h EdU labeling period at either day 1 of adulthood (fog-1) or day 3 of adulthood (wild-type and daf-22) for worms that had been kept at low or high density. For statistical tests see S7B–S7D Table. Asterisks indicate significance of Fisher’s exact test p-value.

Since population density affects a number of aspects of worm physiology [42–44], we next asked whether that density also affects mitotic zone dormancy. Starting from the L2 stage, we kept populations of virgin *fog-1* females either singled (low density) or as group of 70 individuals (high density) on 35 mm plates, and mated them at day 4 of adulthood to assay reproductive capacity. We found that females that had been kept at low density had a significantly reduced brood size compared to those that had been kept at high density (Fig 8A and S7A Table). We then assayed mitotic zone dormancy at day 1 of adulthood, and found a significant effect of population density on that dormancy (Fig 8B and S7B Table): 23% of singled female mitotic zones were dormant vs 46% for the higher-density population. Females thus undergo faster reproductive senescence and cycle more actively when they are singled. To ask whether hermaphrodite dormancy is also density-dependent, we performed the same experiment on selfed hermaphrodites assayed at day 3 of adulthood. We observed a similar effect as for females, with 9% dormancy for singled hermaphrodites vs 27% for the higher-density population (S7C Table). As a first step in elucidating the mechanism underlying this population-density dependence, we repeated the experiment using the *daf-22(m130)* mutation, which abrogates dauer pheromone synthesis [45], and found that density dependence of mitotic zone dormancy was lost (42% vs 47% for high and low densities, respectively; Fig 8B and S7D Table). Mitotic zone dormancy is therefore regulated by population density, most likely through a mechanism that involves dauer pheromone.

Finally, we asked whether intermittent cycling is a behavior that is always associated with reduced overall reproductive activity, or whether it is specific to a state in which worms are well fed but deprived of sperm. Caloric restriction strongly reduces the rate of reproduction as well as total brood size [2,46]. In our hands, hermaphrodites kept in liquid culture with 1x10^10^ bacteria/mL (“high” concentration) had a similar brood size to hermaphrodites kept on solid medium under standard conditions (294 vs. 306; n = 11 and 12, respectively; p > 0.4), but brood size dropped more than two-fold (138; n = 11; p < 1.5E-6) at 1x10^9^ bacteria/mL (“medium” concentration). Further, [46] showed a ~4-fold drop between 1x10^9^ bacteria/mL and 1x10^8^ bacteria/mL (“low” concentration). At day 1 of adulthood, we transferred wild-type adult hermaphrodites to low, medium or high *E*. *coli* concentrations for 24 h, and subsequently performed 1 h continuous EdU labeling. In contrast to mitotic zones from females or older hermaphrodites, those from food-restricted young hermaphrodites were all active (n = 20 each), even at the lowest food concentration. This suggests that intermittent cycling is a specific response to sperm deprivation.

### Sustained cell cycling keeps gonads primed for reproduction

**Fig 9:**
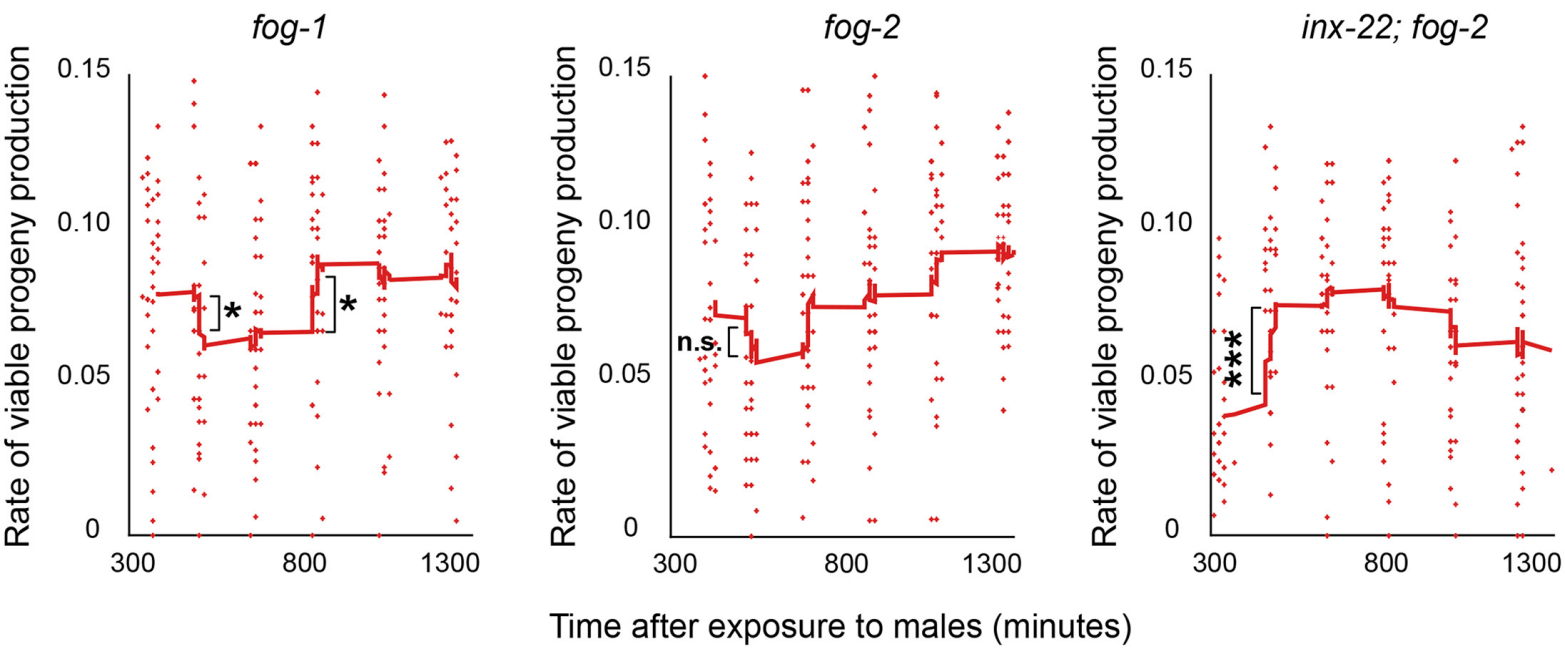
Suboptimal kinetics of response to mating in genotypes and environmental conditions with high gonad dormancy. Early female response to mating on day 3 of adulthood, as assayed by numbers of viable progeny laid per hour. Each point is computed from the number of progeny of one worm over a time window ending at corresponding position on x axis (n = 40 worms per genotype tracked over the time course; some points in the graph overlap). Red lines are moving averages. Error bars represent 83% confidence intervals; asterisks indicate significance of Wilcoxon rank sum test p-value.

If stem cell cycling drives reproductive senescence, why would gonads that are not reproductively active maintain cycling and thus hasten their demise? We hypothesized that gonads that are in the active state are poised for reproduction, and thus respond quickly to a favorable change in environmental conditions. To test this hypothesis we characterized the dynamics of reproduction initiation after mating aged females. After mating of *inx-22; fog-2* females at day 3 of adulthood, the rate of viable progeny production increased sharply and remained sustained over the first ~18 h (Fig 9). By contrast, mated *fog-2* females of the same age experienced a transient increase followed by a trough in progeny production between 9 h and 13 h after mating; this trough was more marked and longer-lasting for *fog-1* females (Fig 9; note that the maximal rates were lower for *inx-22; fog-2* than for *fog-1* or *fog-2*, which is expected given the faster reproductive senescence of *inx-22; fog-2*). The trough likely resulted from a drop in the numbers of developing oocytes in diplotene or diakinesis, which was not experienced by *inx-22; fog-2* (S8A Table and S6A Fig), and appeared to be independent of apoptosis: initial progeny production occurred at the same rate in apoptosis-deficient strains carrying a *ced-3* mutation (S8B Table and S6B Fig), and apoptosis levels decreased within the first 8 h after mating (S8C Table and S6C Fig) and thus likely did not account for a diminished supply of oocytes. In summary, mutant populations that maintain a higher proportion of dormant gonads are less capable of sustaining a high rate of progeny production shortly after mating. Overall, our results are consistent with dormant gonads needing time to make the switch to a state with maximal reproduction rate. Diminished initial reproductive activity of gonads that have been in a dormant state might stem from a detrimental impact of prolonged meiotic arrest on gamete quality or on the gonad region that houses them [47], or from a delay in returning to the active state. In any case, there is strong selection against reproduction delays [5,48]. Avoiding delays provides a powerful rationale against gonads staying fully dormant until reproduction becomes possible.

## Discussion

Our findings significantly extend understanding of *C*. *elegans* reproductive senescence. We showed, using direct experimental manipulation of the cell cycle as well as using comparisons of senescence rates in strains that differ in gonad activity, that activity of the gonad is largely responsible for the progression of reproductive senescence—in particular as a result of germ cell cycling. In the course of this study we discovered that germ cell cycling can be intermittent, which as discussed further below suggests that worm populations may employ an active strategy to manage the progression of reproductive senescence. These results are in contrast to the idea that this progression is solely the result of passive damage accumulation as a function of elapsed time.

What mechanisms tie reproductive senescence to germ cell cycling? Mutations that delay senescence may provide an entry point to start addressing this question. But although a number of mutations have been uncovered that increase reproductive lifespan, most do not result in an increase in total brood size and many in fact result in a *decrease* in brood size—i.e. the reproductive system is less active for a longer period of time, with reduced total output (e.g. [3]). Given our observations that gonad activity drives reproductive senescence, the primary effect of many of these mutations may thus simply be to alter the rate of reproduction, rather than to alter the relationship between reproductive senescence and gonad activity. By contrast, the DDR reduction of function mutant *hus-1(op241)* is to the best of our knowledge the first worm mutant reported to have a total reproductive capacity greater than wild-type. What link between cell cycling and reproductive senescence does this mutant suggest? It is possible that an intact DDR is required for full-speed germ cell cycling, and that *hus-1(op241)* germ cells thus cycle more slowly, leading to delayed reproductive senescence; given that *hus-1(op241)* does not display slower reproduction as might be expected from slower germ cell cycling, we do not favor this possibility. Another possibility is instead that the DDR, whose intensity according to RPA-1 foci correlates positively with the amount of past cell cycling, could mediate the effects of germ cell cycling on reproductive senescence—through molecular pathways that will require further study. An interesting additional problem will be identifying the downsides of increased reproductive output in *hus-1(op241)* mutants. We speculate that late-born progeny are more prone to mutation accrual or genomic rearrangements; further study will be required to characterize the fascinating tradeoff between increased brood size and decreased genome quality in progeny.

We also note that germ cell cycling has been shown by others to shorten lifespan, in addition to reducing reproductive capacity as we report here: mating acts via DAF-12 –a key mediator of lifespan control by the reproductive system [49]–to cause somatic “shrinking” and death, while cell cycle inhibition protects from this effect [50]. It will prove interesting to ask whether the reproductive senescence and somatic lifespan effects of germ cell cycling are enacted by overlapping mechanisms.

The role of germ cell cycling in driving reproductive senescence makes intermittent cycling particularly intriguing. Much of this intermittent cycling phenomenon remains to be characterized. The lack of suitable live imaging techniques makes it necessary to infer the existence of dormant and active states and the rules for transition between these states, and certainly leaves open the possibility that, in the future, more fine-grained studies will identify a broader array of sub-states and more complex transitions between them. The existence of at least two states, an active and a dormant one, is strongly supported by EdU continuous labeling studies that show that some virgin females mitotic zones label immediately when placed on EdU while some do not. This is compatible with germ cells being capable of stopping replication within S-phase or significantly lengthening S-phase [51,52]. Further, analysis of mitotic index CV shows greater variability between mitotic zones of virgin female populations than between those of mated female or young hermaphroditic populations. This is compatible with the existence of at least two cell cycle states—dormant and active—but there could be a continuum of intermediary states, whose practical relevance may depend on the amount of time they perdure.

Until cell cycle states are more finely defined at the molecular level, control of transitions between states can only be addressed in qualitative terms. The spread in virgin female mitotic zone cell cycle progression during EdU pulse-chase experiments (Fig 6C) suggests that the transition between dormant and active states has a stochastic aspect (although we cannot formally exclude that individuals possess strongly intrinsic differences in switching rates); this is also suggested by the larger reproductive capacity CV in virgin females mated at day 2 than in those mated at day 0, which likely stems from a random distribution of time between days 0 and 2 spent in the active state that reduces reproductive capacity. We note however that biological phenomena are often described as “stochastic” until a deterministic underlying is identified; for example, control of the lysis-lysogeny decision of the lambda phage was described as stochastic but cell volume turned out to be a strong predictor of cell fate [53]. The switch between actively-cycling and dormant states could thus be downstream of an unknown highly-deterministic mechanism rather than being controlled by molecular noise. But in any case stochastic switching between active and dormant states provides a fitting and parsimonious model for our current data.

The molecular controls of intermittent cycling remain to be fully established. It may seem surprising that *fog-1* and *fog-2*, both known for their role in germline sex determination, have different cell cycle dynamics: *fog-1* undergoes slower average cell cycling that does *fog-2*. This could be consistent with the previously-established, dose-dependent role of FOG-1 in promoting proliferation [54]. But a complication in comparing *fog-1* and *fog-2* exists in that virgin *fog-1* ovulates at a slower rate than virgin *fog-2;* it is possible that a slower loss of cells to oogenesis leads to slower homeostatic mitotic zone cycling, rather than there being a direct role for *fog-1* in controlling cell cycling. In line with this idea, it has most interestingly been shown recently that oocyte accumulation inhibits germ cell proliferation [41]. Lastly, *fog-1* and *fog-2* differ in the foraging behaviors [55], which could impact germ cell cycling. Overall, further experiments will be required to address the different behavior of *fog-1* and *fog-2*.

In addition to the *fog-1* and *fog-2* genes better known for their role in germline sex determination, our results and those from another recent study [41] implicate a number of genes and pathways in control of proliferation in virgin female mitotic zones. We have identified a role played by *daf-22*, likely through the dauer pheromone pathway, while the *daf-18* and insulin pathways were implicated by [41] (in a way that did not address dormant and active states but that is compatible with our results when considering average cell cycle speed). Further experiments will be required to derive a more comprehensive understanding of the integration of these molecular controls.

What is the relevance of intermittent cycling? First, we address whether it is a general response to a reduced need for germ cell production. A germline proliferation stop occurs quickly in females after deprivation of food [51,52]. In cases where a reduced average proliferation rate is warranted, different possibilities would be for mitotic zones to be reduced in size, for them to cycle at a slower but steady rate, or for them to go through periods of dormancy. We have not observed substantial changes in mitotic zone size. We did observe changes in cell cycle length across developmental stages: we recently reported that germ cells cycle ~60% more slowly in young adult hermaphrodites than in L4 hermaphrodites, in a way that does not involve intermittent cycling but rather S-phase lengthening [34]; we have also shown in the present study that caloric restriction that reduces the rate of reproduction over 2-fold also does not result in intermittent cycling. Therefore, intermittent cycling is a response that so far appears specific to well-fed females and hermaphrodites with a diminished sperm supply.

Next, we address whether intermittent cycling may occur in the wild in a fashion that is relevant to “fitness.” We focused largely on genetically-feminized *C*. *elegans* hermaphrodites in the present study, because *C*. *elegans* has been more thoroughly studied than other species and can be investigated with better established genetic tools. Although *C*. *elegans* females do not occur naturally, we showed that the same intermittent cycling behavior occurs in *C*. *remanei*, which is a gonochoristic (i.e. male/female) species, and in older hermaphrodites.

What is the relevance of germ cell behavior in older, sperm-depleted hermaphrodites? Two important questions to address this relevance are 1) how strong a selective pressure applies to hermaphrodites around the end of reproduction and 2) whether hermaphrodite mating is of much relevance in a mostly selfing species. We discuss these two questions in turn. Regarding whether reproduction of older hermaphrodites is under active selection, there is a general expectation that late-life reproduction might only be under weak selection ([56]; although see e.g. [57] for recent developments). Interestingly however, hermaphrodites show mating behavior that is specific to old age. Specifically, *older* hermaphrodites release a volatile pheromone that attracts males [58], which is regulated by the CEH-18 sperm sensing pathway [59]. In addition, older hermaphrodites mate more rapidly with males and eject sperm with reduced frequency than young hermaphrodites [60]. If evolutionary pressures were such that behavior of older hermaphrodites was of little relevance to fitness, one would expect that mutation accumulation would have caused these old-age specific behaviors to be lost [see e.g. 61]. It may of course be that these behaviors are just a remnant of a not-too-distant gonochoristic past. But in that case, the intermittent cycling behavior we observe would likely also be preserved from the previous gonochoristic state and thus amenable to study in *C*. *elegans—*even if it has lost its direct purpose. Overall, hermaphrodite mating that occurs under conditions of intermittent cycling is likely of relevance to fitness and is thus under active selection, or was in a suitably-recent past.

Regarding the general in-the-wild relevance of outcrossing (irrespective of hermaphrodite age at the time of mating), although *C*. *elegans* males have only been isolated at a very low frequency in the wild [62,63] heterozygosity data show that outcrossing does occur in the wild—crucially, at a higher frequency than expected from mere spontaneous generation of males through meiotic non-disjunction of the X chromosome [reviewed by 61]. Males thus appear to be actively maintained in the wild. Consistent with this, experiments in the laboratory have identified conditions likely to be encountered in the wild, such as increased mutational pressure or exposure to changing environments, that lead to maintenance of males at a high frequency [61,64]. Lower than expected observed frequencies in the wild may be due to outbreeding depression [61], and male frequency may thus be actively maintained at an equilibrium frequency by counteracting evolutionary forces. Taken in combination with the fact that older hermaphrodites are more likely than young hermaphrodites to have cross progeny with males, this strengthens the idea that intermittent cycling in older hermaphrodites is a behavior that is of relevance in the wild and actively selected for, even if that selection is weaker than the selection for early hermaphroditic reproduction.

If future reproduction of intermittently-cycling hermaphrodites is relevant to in-the-wild conditions and is specifically selected for, how would intermittency be beneficial? We speculate that uncertainty in the time at which reproduction will become possible could perhaps underlie a bet-hedging strategy. Bet-hedging is a well-established strategy followed by unicellular and multicellular organisms to avoid or spread risks in the face of uncertain environmental conditions [65,66], and phenotypic heterogeneity is a mechanism by which bet hedging can be implemented [67]. Specifically, a model is conceivable whereby in unfavorable conditions *C*. *elegans* populations hedge their bets by maintaining individuals that are primed for reproduction at the cost of faster senescence, and individuals whose gonads are dormant and that thus senesce more slowly, helping preserve reproductive capacity of the population over time. Stochasticity in the behavior of individual gonads would then have two roles. First, it would allow individuals to modulate the average rate of germ cell cycling. Second, and more interestingly, stochasticity is a parsimonious mechanism to develop a broad distribution of effective reproductive senescence rates in the population, without those rates being pre-assigned to each individual. Individuals that “take a chance” by cycling allow the population to quickly initiate reproduction if conditions become favorable shortly after they cycle, while individuals that stay in a dormant state avoid the population going extinct if conditions only become favorable after an extended period of time (during which individuals that frequently cycled exhausted their reproductive capacity). To our knowledge, such bet hedging would be the first to directly control senescence of a self-renewing organ, and the first to be implemented by integration of stochastic switching between different states. An interesting problem for future studies would be to ask whether bet-hedging strategies are used by other self-renewing organs in which stem cell cycling contributes to maintenance of tissue function, but increases the probability of a cell becoming senescent to avoid cancer [68].

Finally, we note that there may be a number of alternative reasons for intermittent cycling. Intermittent cycling may be an unselected-for, side-effect behavior that derives from the particular structure of the yet-to-be-characterized gene network that regulates it, while the increase in phenotype variability in old age could be explained by phenotypic drift [69]. Rather than implementing a bet hedging strategy, intermittent cycling could provide a convenient means for cells to alternate between different metabolic states, or could be a way of maintaining the same distribution of cell cycle phases in the mitotic zone irrespective of the average cycling rates—so that mitotic zones can assume an optimal distribution quickly when reproduction initiates. Future studies will be required to explore these ideas.

## Materials and Methods

### Worm strains and maintenance

Strains used were Bristol N2, JK3743: *fog-1*(*q785*) I [11], JK1078: *fog-2*(*q71*) V [6], XM1012: *inx-22*(*tm1661*) I; *fog-2*(*q71*) V [9], EM464: *C*. *remanei* [36], BA784: *spe-8(hc50)* I [13], CNQ41: *fog-1(q785)* I*; ced-1*::*gfp(bcIs39)* V (obtained by a cross of MD701 [70] and JK3743), CNQ44: *ced-3(n717)* IV*; fog-2(q71)* V (obtained by a cross of MT1522 [71] and JK1078), WS4581: *rpa-1*::*yfp(opIs263)* [28] and WS2277: *hus-1(op241)* I [31].

Strains were maintained as described [72] using *E*. *coli* HB101 as a food source. Worms were staged by picking at the L4 stage as identified by visual inspection of vulva shape. Unless otherwise specified, 50 worms were kept per 60 mm plate prior to mating. Mating at “day 0” of adulthood refers to exposure to males for 24 h starting from L4—so that females are exposed to males at the earliest time at which they are sexually mature. More generally, mating at day n of adulthood refers to exposure to males from L4 + n * 24 h to L4 + (n+1) * 24 h. For mating and cell cycle experiments, a sharp increase in cycling intermittency was noted in wild-type selfed hermaphrodites around day 3 adulthood. Therefore, when using day 3 hermaphrodites, special care was taken to use worms at precisely L4 + 72 h. Female mating was performed on 35 mm-diameter plates (CC7672-3340, USA Scientific, Ocala, FL), at a density of 1 female and 3 young adult males per plate. For *C*. *remanei* matings, females were continuously exposed to males, which were refreshed every three days; without this continuous exposure, female reproductive capacity is not exhausted (progeny count with 24 h male exposure was 288, n = 20, compared with 734, n = 20, with continuous exposure). For brood size scoring, mothers were passaged every day to 35 mm fresh plates, until the end of reproductive activity. Plates on which embryos had been laid were incubated for ~2 days to let progeny hatch and grow in size; progeny were counted before they reached the adult stage. Mothers that crawled off agar were censored from the analysis.

To analyze the early response to mating in fine detail, females isolated 3 days after L4 were each plated with 3 males and any progeny removed from the plate every ~3 h for a total of 21 h. After another 24 h, transferred progeny were scored for viability.

### Cell cycle arrest

For cell cycle inhibition experiments, hydroxyurea (HU) or the CDK inhibitor Roscotivine (H8627 and R7772, respectively Sigma-Aldrich, St. Louis, MO) were freshly prepared and added to NGM-agar (cooled to 55°C prior to dispensing) at a final concentration of 40 mM for HU [73] or 50 μM for Roscovitine. Roscovitine was diluted in DMSO so that the final dilution of the solvent in NGM was 1:1000; control plates were also supplemented with DMSO at 1:1000. The following day, bacteria were UV-killed by placing open seeded plates in a Spectrolinker XL-1500 (Spectroline, NY) for 5 min, and then transferred onto HU, Roscovitine or control plates. Immediately after, 1-day adult virgin females were transferred to HU or control plates for 24 h. They were then returned to regular plates and mated as above.

For starvation experiments, females were picked at the late L4 stage, rinsed 5 times in M9, and starved in complete S-medium [74]. Following starvation, females were transferred to seeded plates, on which they were kept for 24 h prior to mating.

### Thermotolerance and lifespan assays

For the thermotolerance assay, worms were transferred to HU or control plates at day 1 of adulthood, kept on these plates for 24 h, returned to regular plates, and shifted to 35°C. Every 2 h until all worms had died, plates were removed individually from the incubator and the number of live and dead worms recorded.

For the lifespan assay, worms were initially treated as for the thermotolerance assay but without temperature upshift. A count was made of live and dead worms every day until all worms had died. Worms that had desiccated on the side of plates or died due to an exploding vulva were censored from the time of death.

### Dietary restriction

Worms were kept on NGM plates until day 1 of adulthood, at which point they were transferred to 500 μl S-medium containing serial dilutions of HB101 at final concentrations of 10^8^ (low), 10^9^ (medium) or 10^10^ (high) cells / mL in 24-well tissue culture plates rocked on a nutator. At day 2 of adulthood worms were pulsed for 1 h with EdU resuspended in water and delivered at a final concentration of 0.4 mM and processed as detailed below.

### Staining and imaging

To label cells with the thymidine analogue EdU (C10337, Life Technologies, Grand Island, NY), worms were fed EdU-labeled *E*. *coli*. To prepare labeled *E*. *coli*, strain MG1693 was grown in minimal medium supplemented with glucose [75] and 75 μM EdU for pulse-chase experiments and 7.5 μM for continuous labeling experiments. When required, Fluoresbrite fluorescent microspheres (19507–5, Polysciences, Warrington, PA) were added to bacteria prior to seeding, at a 1:100 dilution. Immediately following seeding, plates were stored at 4°C. Plates were warmed to 20°C prior to use. Worms were kept for 0.5–8 h on EdU-labeled bacteria in the dark, returned to non-labeled bacteria for experiments that required a chase, and were fixed and processed as described [76] using 0.1 μg/ml DAPI to label DNA and 1:200 anti-PH3 antibody (9706, Cell Signaling, Beverly, MA) to label M-phase cells, and imaged at ~0.3 μm z intervals with LSM 710 or 780 confocal microscopes (Carl Zeiss MicroImaging, Oberkochen, Germany), using a 63x objective.

EdU continuous labeling image data were assayed manually for the presence of at least one EdU-positive cell in individual mitotic zones. EdU pulse chase data were analyzed as described in S1 Text.

For whole germ line imaging, for counts of apoptotic cells detected with the CED-1::GFP reporter or foci detected with the RPA-1::YFP reporter, and for counts of cells in diplotene or diakinesis extruded germ lines were fixed and stained with DAPI, 0.16 μM Alexa 594-conjugated Phalloidin (A12381, Life Technologies), and 1:1000 anti-GFP (for CED-1::GFP and RPA-1::YFP; ab5450, Abcam, Cambridge, MA). Image stacks were acquired at 0.3–0.6 μm z intervals using a 63x objective. In some instances, several panels were imaged for each gonadal arm that were subsequently stitched [77].

### Quantification of RPA-1 foci

RPA-1 foci were analyzed in the proximal meiotic region (from the beginning of zone 5 and into 6, as described [78]). The dataset was blinded by a user who copied renamed files from all datasets into a single directory. A second user scored each of the renamed image files by selecting 20 random cells in zones 5 and 6 using the DNA channel of the image and recording RPA-1::YFP foci within each of these cells using Parismi [52].

### Statistics

The Wilcoxon rank sum test as implemented by the R project or Matlab was used to test for significance of differences (two-tailed test), unless otherwise stated. A generalization to interval-censored data of the Wilcoxon test implemented by the “interval” R package [79] was used to analyze labeling times in continuous EdU labeling experiments. Bootstrapping was performed using the “boot.ci” function of the “boot” R package [80]. The log-rank test was used for analysis of survival data.

## Supporting Information

S1 Fig Effect of genotype on reproductive capacity. Remaining reproductive capacity when mated at day 2 of adulthood as a function of capacity when mated at day 0. There is a significant main effect of genotype on reproductive capacity between all genotype pairs except *inx-22; fog-2* and *spe-8*. For numbers and statistical tests see S1B Table.
(TIF)

S2 Fig Number of apoptotic cells identified using a *fog-1; ced-1*::*gfp* reporter strain. For numbers and statistical tests see S2B and S2D Table.
(TIF)

S3 Fig Absence of detectable hormesis in HU-treated females. (A–B) HU-treated females have reduced survival in both lifespan (A) and thermotolerance (B) assays. Asterisks indicate significance of p-value computed by applying a log-rank test to the survival curves. For numbers and statistical tests see S3C Table.
(TIF)

S4 Fig Characterization of stochastic cycling. (A) Worms ingest food irrespective of reproductive activity. Overlay of transmitted light and fluorescence signal (green), showing bead ingestion by *fog-2* females after 1 h exposure (n = 30). (B) Older, wild-type hermaphrodites display stochastic cycling with dynamics close to those of *C*. *elegans* females. This is rescued upon mating. Graph shows fractions of mitotic zones remaining unlabeled as a function of time on EdU-labeled food (n = 40–55 for each time point). (C—D) *C*. *remanei* females undergo reproductive senescence and stochastic cycling similar to *C*. *elegans* females. (C) The brood size of *C*. *remanei* females mated at days 0, 3, and 7 of adulthood (n = 15–20 for each time point). Error bars represent 83% confidence intervals; asterisks indicate significance of Wilcoxon rank sum test p-value. (D) Fractions of mitotic zones remaining unlabeled as a function of time on EdU-labeled food for virgin or mated *C*. *remanei* (n = 40–50 for each time point). (E) Oocyte laying counts for *fog-1* or *fog-2* females from day 0 to day 6 of adulthood. *fog-2* lays significantly more oocytes than *fog-1* (p < 0.04, n = 35 for *fog-1* and 31 for *fog-2)*.
(TIF)

S5 Fig Cell cycle analysis. (A–C) Overview of image processing for cell cycle analysis (reproduced in part from [52]). (A) Sections across xy, xz, and yz planes of a three-dimensional image of a gonadal arm, showing DNA (green) and EdU (overlaid in red) after a pulse with no chase. (B) End product of image segmentation for gonadal arm shown in G. Blue circles show cell outlines computed by our pipeline. (C) Scatterplot of DNA/EdU contents. Each point is a cell from the gonadal arm shown in G and H. Color shows cycle phase automatically assigned after thresholding of DNA and EdU contents. This particular gonadal arm was chosen for its clarity; see S1 Dataset for full set of wild-type data. (D–F) Overview of process to assay cycle progression of individual mitotic zones. (D) Simulated cell cycling in a mitotic zone after an EdU pulse followed by chase. Each set of histograms shows DNA content of EdU-positive cells (blue) and EdU-negative cells (red), at a range of times shown as percentage of cell cycle completion (0% corresponds to time of EdU pulse, and 100% to all cells in mitotic zone having undergone a full cycle during the chase and the histograms thus having returned to their original state). A subset of the 20 time points that were computed is shown. See S1 Movie for animated histograms. (E) Comparison of simulated DNA content histograms (top row) with experimental histograms for which they provided the best fit according to the Earth Mover's Distance. To provide a representative view of the experimental data, for each cell cycle completion column we show the mitotic zone that gave the best, median, or worst fit in its category to simulated data. A full set of histograms is shown in S1 Dataset. (F) Rationale for using a circular Earth Mover's Distance, shown on theoretical histograms. Histograms (i) and (iv) are close in time but are separated by a long distance unless the x axis wraps around from 2n to 1n.
(TIF)

S6 Fig Changes in older females after mating. (A) Number of cells in diplotene (red), and number of cells in diakinesis (green) for *fog-1* and *inx-22; fog-2* at times after mating corresponding to the trough in *fog-1* progeny production (black lines at top show rate of viable progeny production; see Fig 9 for details). For numbers and statistical tests see S8A Table. (B) Number of progeny produced within the first 12 h after mating *fog-2* or *ced-3; fog-2* females on day 3 of adulthood. For numbers and statistical tests see S8B Table. (C) Numbers of germ line apoptotic cells after mating *fog-1; ced-1*::*gfp* females on day 3 of adulthood as assayed by CED-1::GFP highlighting of engulfed cells. For numbers and statistical tests see S8C Table. Error bars represent 83% confidence intervals; asterisks indicate significance of Wilcoxon rank sum test p-value.
(TIF)

S1 Table Statistical test results. Associated with Fig 1.
(PDF)

S2 Table Measurements and statistical test results. Associated with Fig 2.
(PDF)

S3 Table Measurements and statistical test results. Associated with Fig 3.
(PDF)

S4 Table Statistical test results. Associated with Fig 4.
(PDF)

S5 Table Initial rates of cell cycle progression after EdU labeling, quality of underlying fits, and other characteristics of cycling cells. Associated with Fig 6.
(PDF)

S6 Table Confidence intervals for brood size CVs. Associated with Fig 7.
(PDF)

S7 Table Statistical test results. Associated with Fig 8.
(PDF)

S8 Table Measurements and statistical test results. Associated with Fig 9.
(PDF)

S1 Dataset DNA content histograms for EdU-positive (blue) and EdU-negative (red) cells in single wild-type gonads at day 1, after an EdU pulse and a 0 h—8 h chase. Numbers displayed on top of histogram are formatted as “gonad_ID (estimated fraction of cell cycle completion)”.
(PDF)

S1 Movie Simulated cell cycling in a mitotic zone during EdU chase. Each set of histograms shows DNA content of EdU-positive cells (blue) and EdU-negative cells (red). The movie consists of two repeats of a whole cell cycle period, and shows how DNA content histograms cycle as cells increase in DNA content as a result of S phase, and return to low DNA contents after division.
(MOV)

S1 Text Cell cycle analysis and computational simulations. (PDF)
