# Supplementary figures and images for: Intermittent Stem Cell Cycling Balances Self-Renewal and Senescence of the C. elegans Germ Line
Source: PLoS Genet. 2016 Apr 14;12(4):e1005985. doi: 10.1371/journal.pgen.1005985 (PMC4831802; doi:10.1371/journal.pgen.1005985)

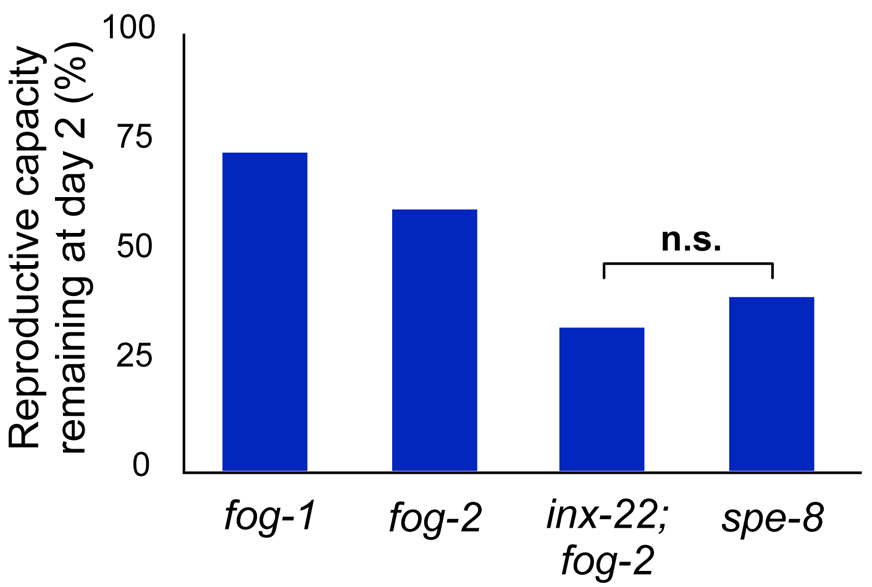

Supplement: S1 Fig — Remaining reproductive capacity when mated at day 2 of adulthood as a function of capacity when mated at day 0. There is a significant main effect of genotype on reproductive capacity between all genotype pairs except inx-22; fog-2 and spe-8. For numbers and statistical tests see S1B Table. (TIF) [file pgen.1005985.s001.tif]

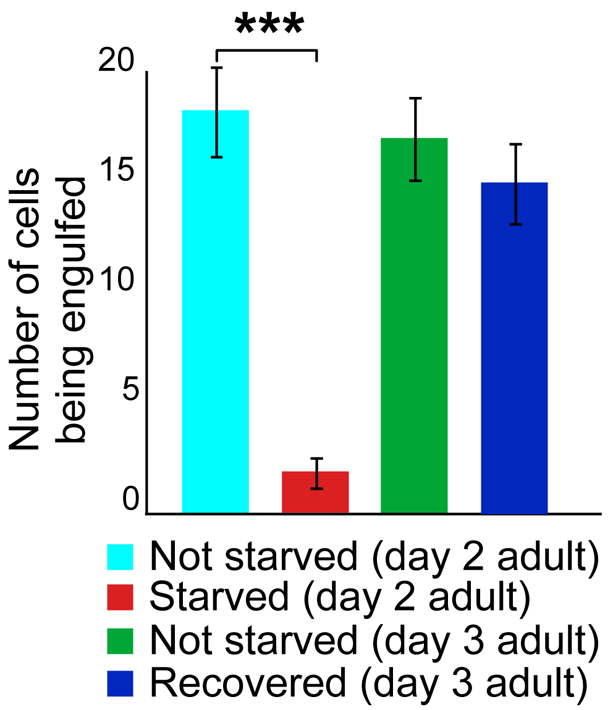

Supplement: S2 Fig — For numbers and statistical tests see S2B and S2D Table. (TIF) [file pgen.1005985.s002.tif]

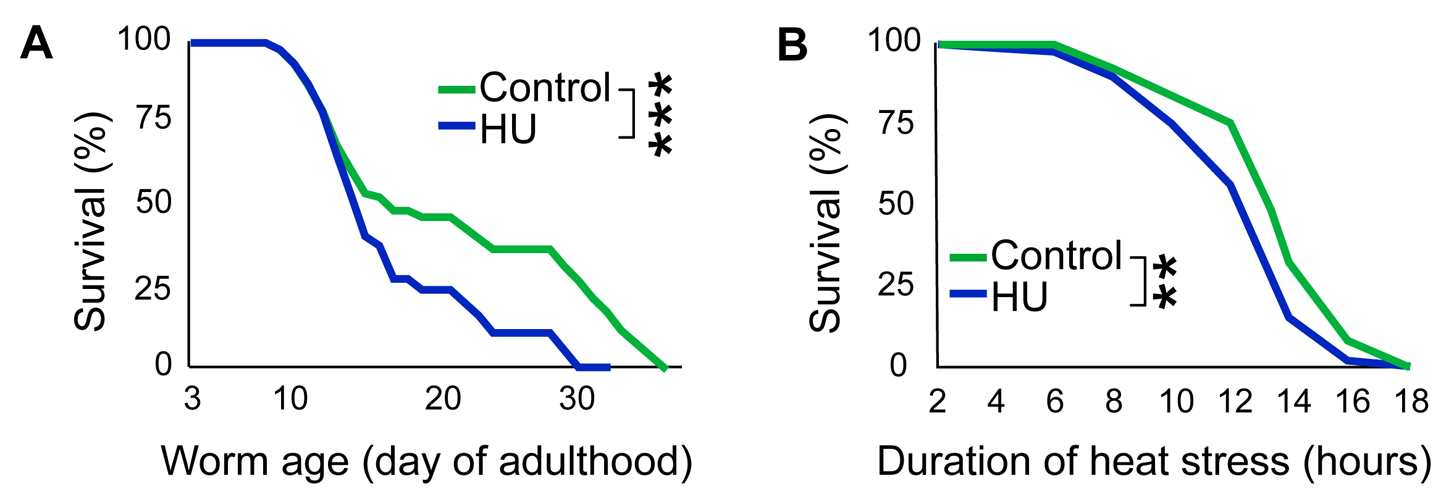

Supplement: S3 Fig — (A–B) HU-treated females have reduced survival in both lifespan (A) and thermotolerance (B) assays. Asterisks indicate significance of p-value computed by applying a log-rank test to the survival curves. For numbers and statistical tests see S3C Table. (TIF) [file pgen.1005985.s003.tif]

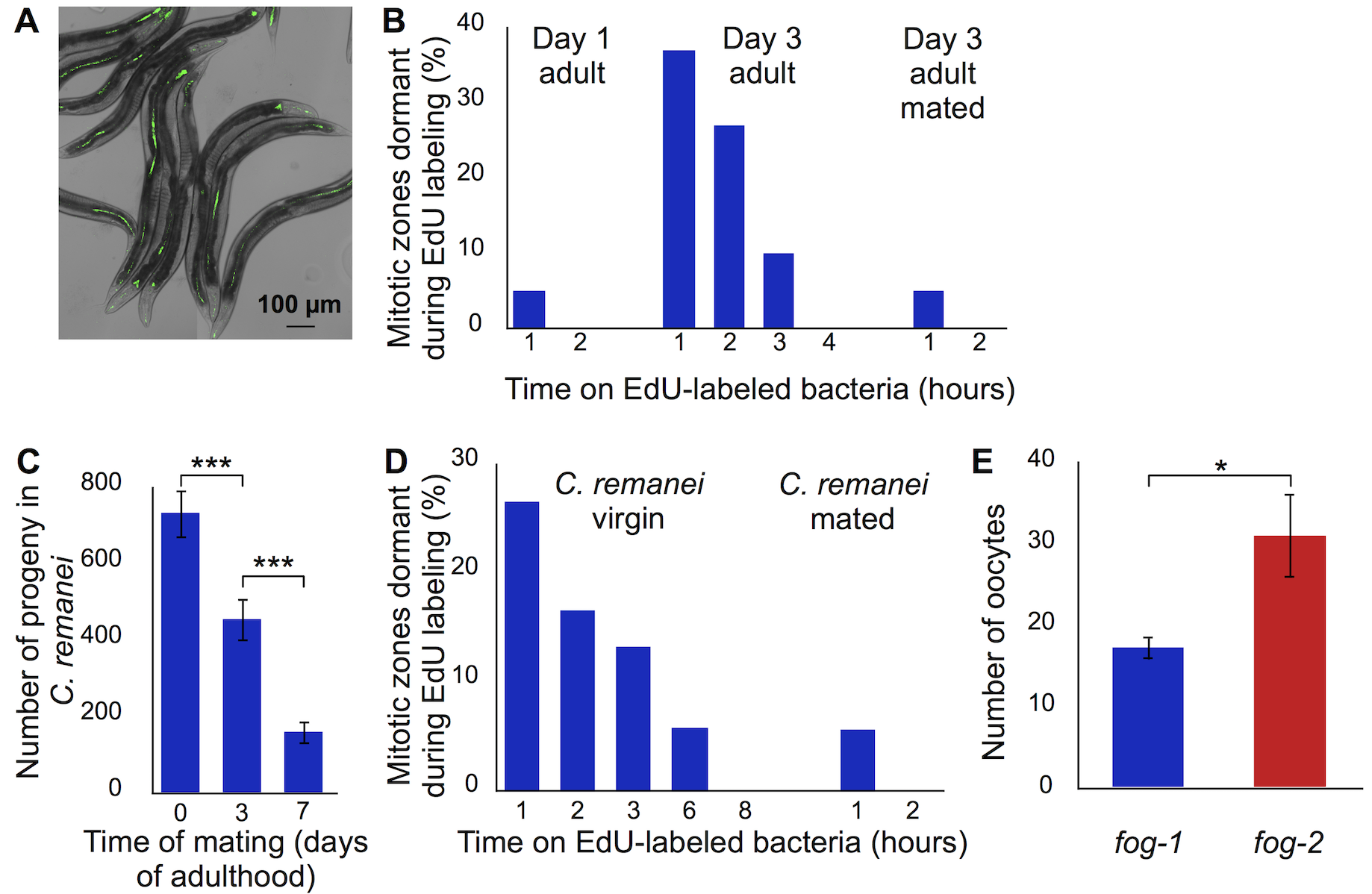

Supplement: S4 Fig — (A) Worms ingest food irrespective of reproductive activity. Overlay of transmitted light and fluorescence signal (green), showing bead ingestion by fog-2 females after 1 h exposure (n = 30). (B) Older, wild-type hermaphrodites display stochastic cycling with dynamics close to those of C. elegans females. This is rescued upon mating. Graph shows fractions of mitotic zones remaining unlabeled as a function of time on EdU-labeled food (n = 40–55 for each time point). (C—D) C. remanei females undergo reproductive senescence and stochastic cycling similar to C. elegans females. (C) The brood size of C. remanei females mated at days 0, 3, and 7 of adulthood (n = 15–20 for each time point). Error bars represent 83% confidence intervals; asterisks indicate significance of Wilcoxon rank sum test p-value. (D) Fractions of mitotic zones remaining unlabeled as a function of time on EdU-labeled food for virgin or mated C. remanei (n = 40–50 for each time point). (E) Oocyte laying counts for fog-1 or fog-2 females from day 0 to day 6 of adulthood. fog-2 lays significantly more oocytes than fog-1 (p < 0.04, n = 35 for fog-1 and 31 for fog-2). (TIF) [file pgen.1005985.s004.tif]

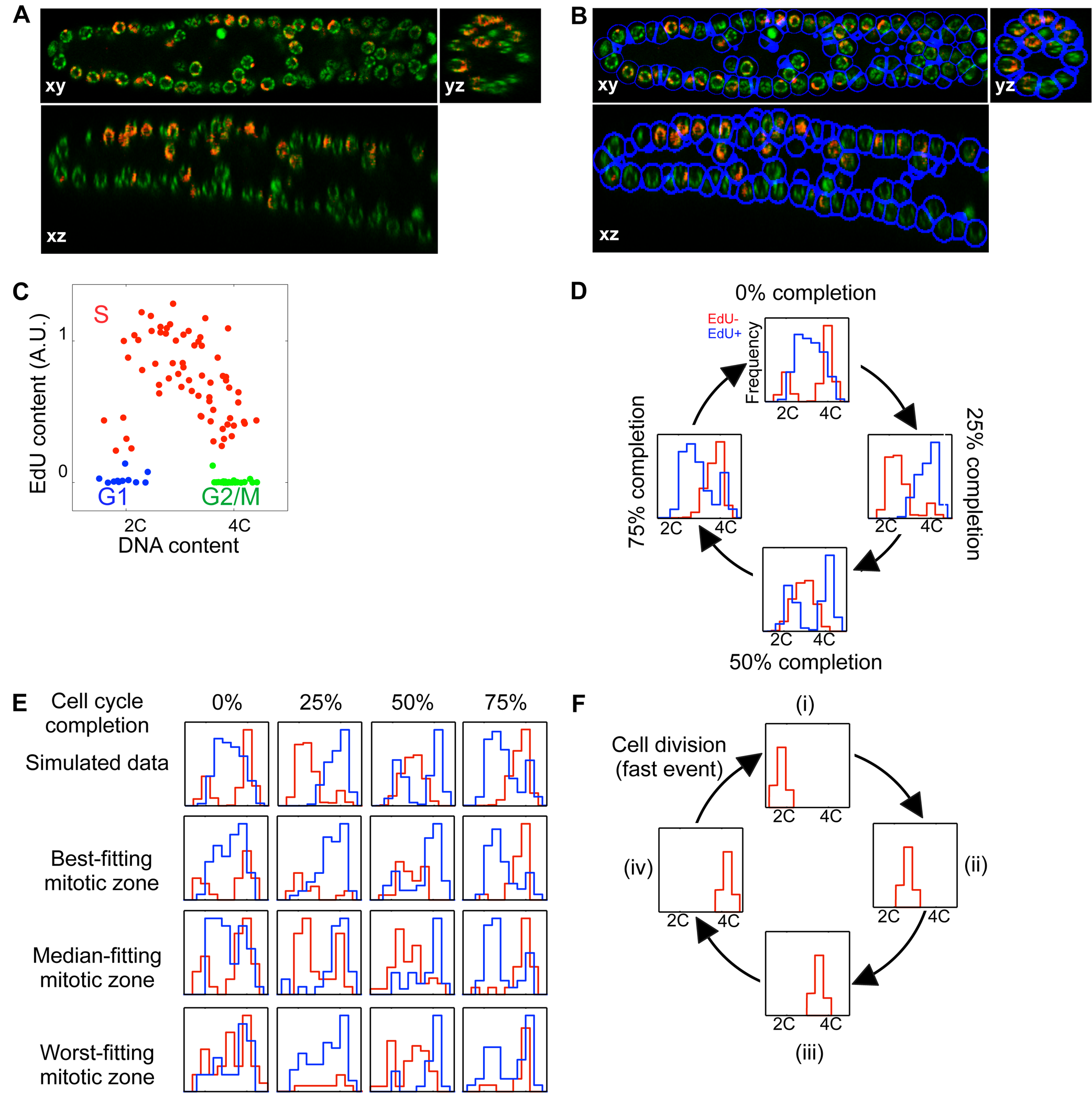

Supplement: S5 Fig — (A–C) Overview of image processing for cell cycle analysis (reproduced in part from [52]). (A) Sections across xy, xz, and yz planes of a three-dimensional image of a gonadal arm, showing DNA (green) and EdU (overlaid in red) after a pulse with no chase. (B) End product of image segmentation for gonadal arm shown in G. Blue circles show cell outlines computed by our pipeline. (C) Scatterplot of DNA/EdU contents. Each point is a cell from the gonadal arm shown in G and H. Color shows cycle phase automatically assigned after thresholding of DNA and EdU contents. This particular gonadal arm was chosen for its clarity; see S1 Dataset for full set of wild-type data. (D–F) Overview of process to assay cycle progression of individual mitotic zones. (D) Simulated cell cycling in a mitotic zone after an EdU pulse followed by chase. Each set of histograms shows DNA content of EdU-positive cells (blue) and EdU-negative cells (red), at a range of times shown as percentage of cell cycle completion (0% corresponds to time of EdU pulse, and 100% to all cells in mitotic zone having undergone a full cycle during the chase and the histograms thus having returned to their original state). A subset of the 20 time points that were computed is shown. See S1 Movie for animated histograms. (E) Comparison of simulated DNA content histograms (top row) with experimental histograms for which they provided the best fit according to the Earth Mover's Distance. To provide a representative view of the experimental data, for each cell cycle completion column we show the mitotic zone that gave the best, median, or worst fit in its category to simulated data. A full set of histograms is shown in S1 Dataset. (F) Rationale for using a circular Earth Mover's Distance, shown on theoretical histograms. Histograms (i) and (iv) are close in time but are separated by a long distance unless the x axis wraps around from 2n to 1n. (TIF) [file pgen.1005985.s005.tif]

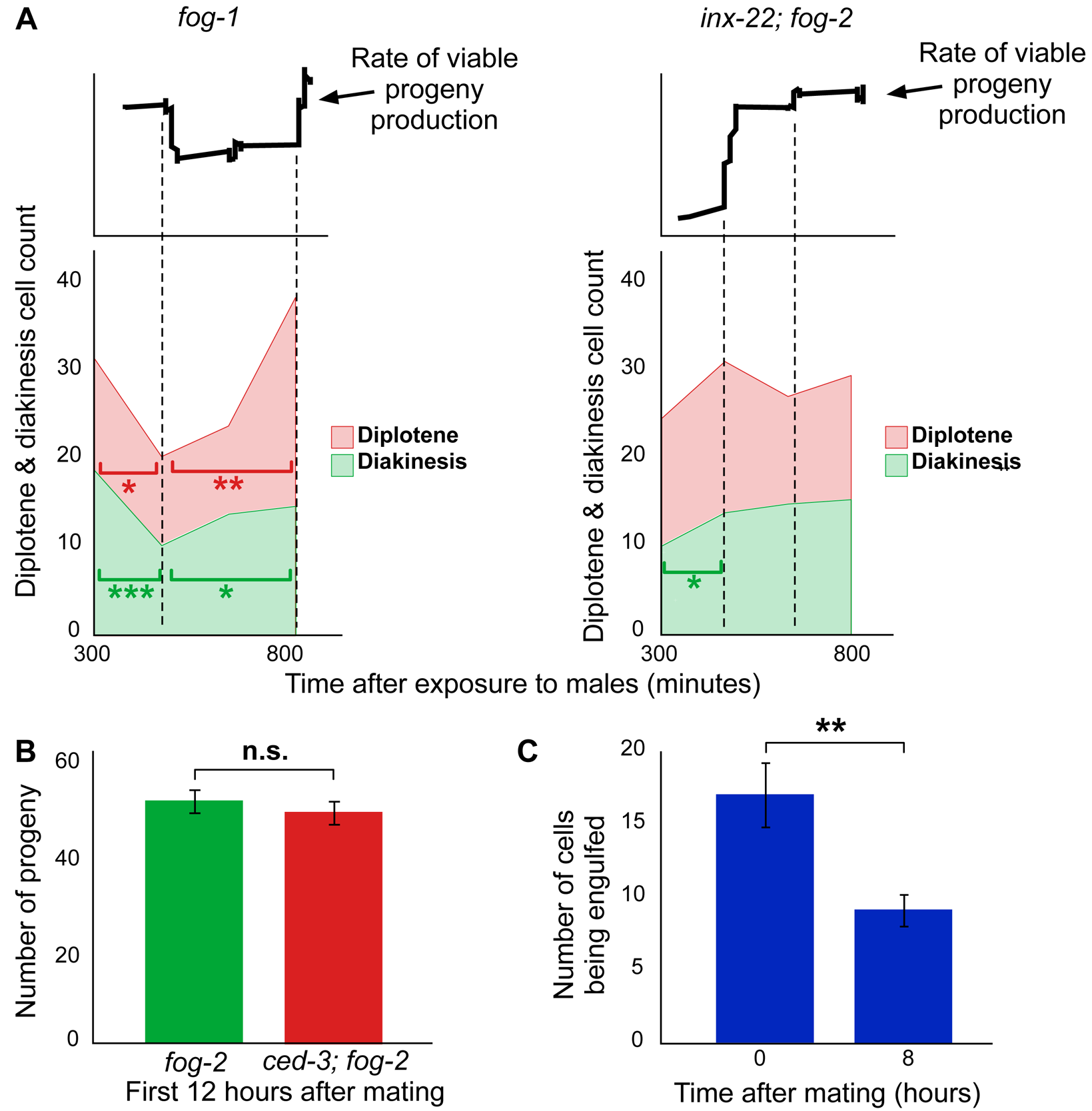

Supplement: S6 Fig — (A) Number of cells in diplotene (red), and number of cells in diakinesis (green) for fog-1 and inx-22; fog-2 at times after mating corresponding to the trough in fog-1 progeny production (black lines at top show rate of viable progeny production; see Fig 9 for details). For numbers and statistical tests see S8A Table. (B) Number of progeny produced within the first 12 h after mating fog-2 or ced-3; fog-2 females on day 3 of adulthood. For numbers and statistical tests see S8B Table. (C) Numbers of germ line apoptotic cells after mating fog-1; ced-1::gfp females on day 3 of adulthood as assayed by CED-1::GFP highlighting of engulfed cells. For numbers and statistical tests see S8C Table. Error bars represent 83% confidence intervals; asterisks indicate significance of Wilcoxon rank sum test p-value. (TIF) [file pgen.1005985.s006.tif]

## 0 hr chase

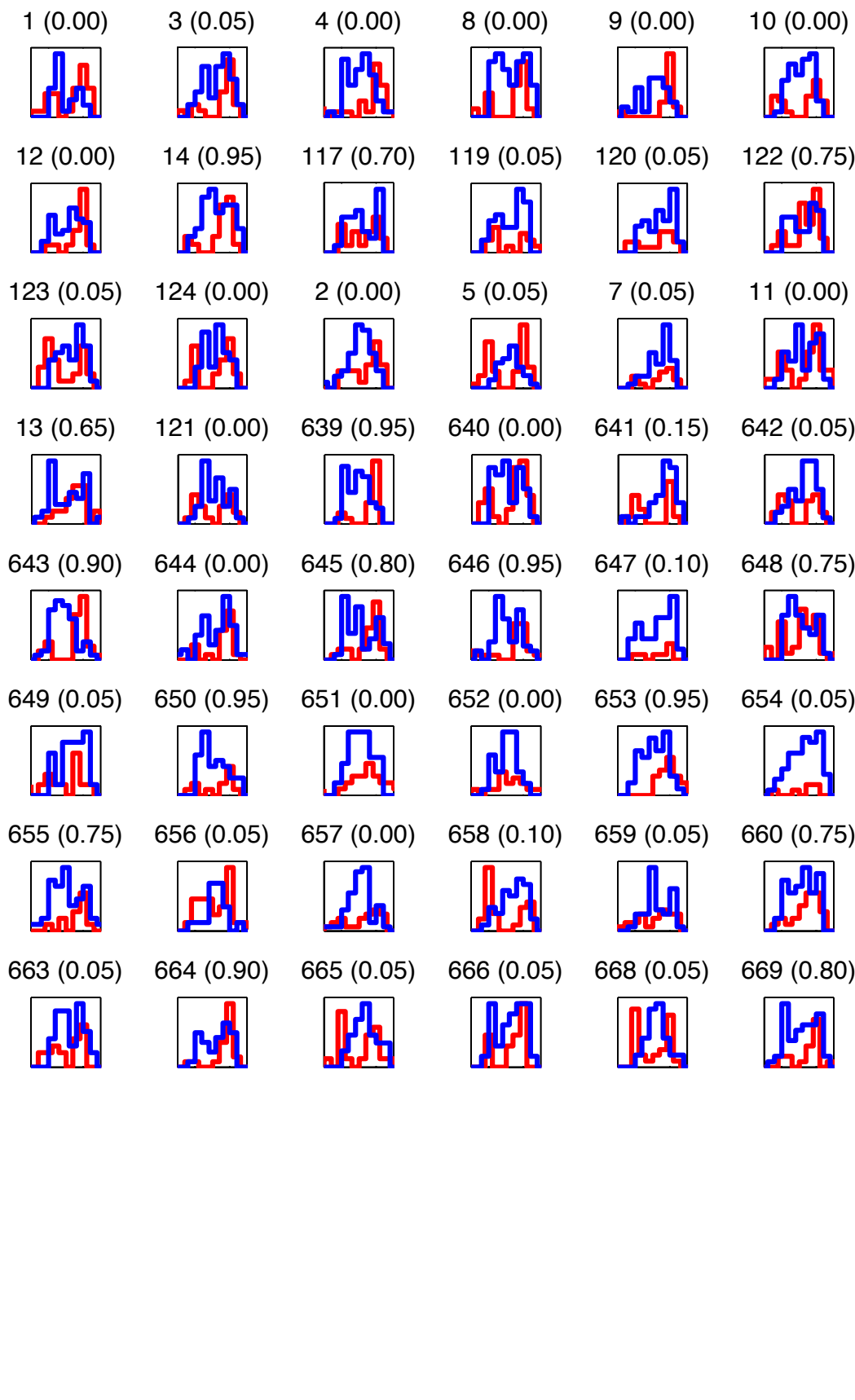

## 2 hr chase

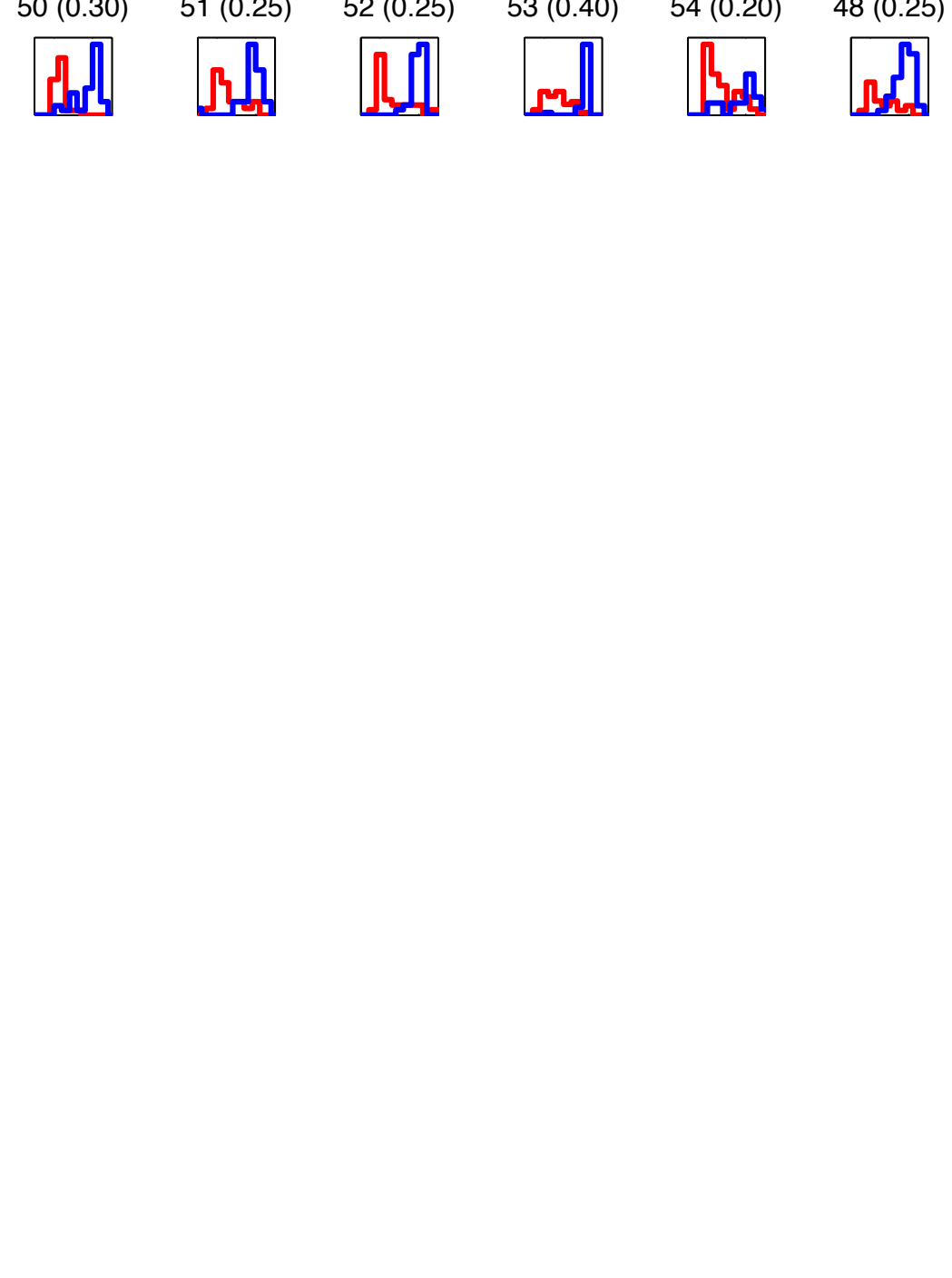

## 3 hr chase

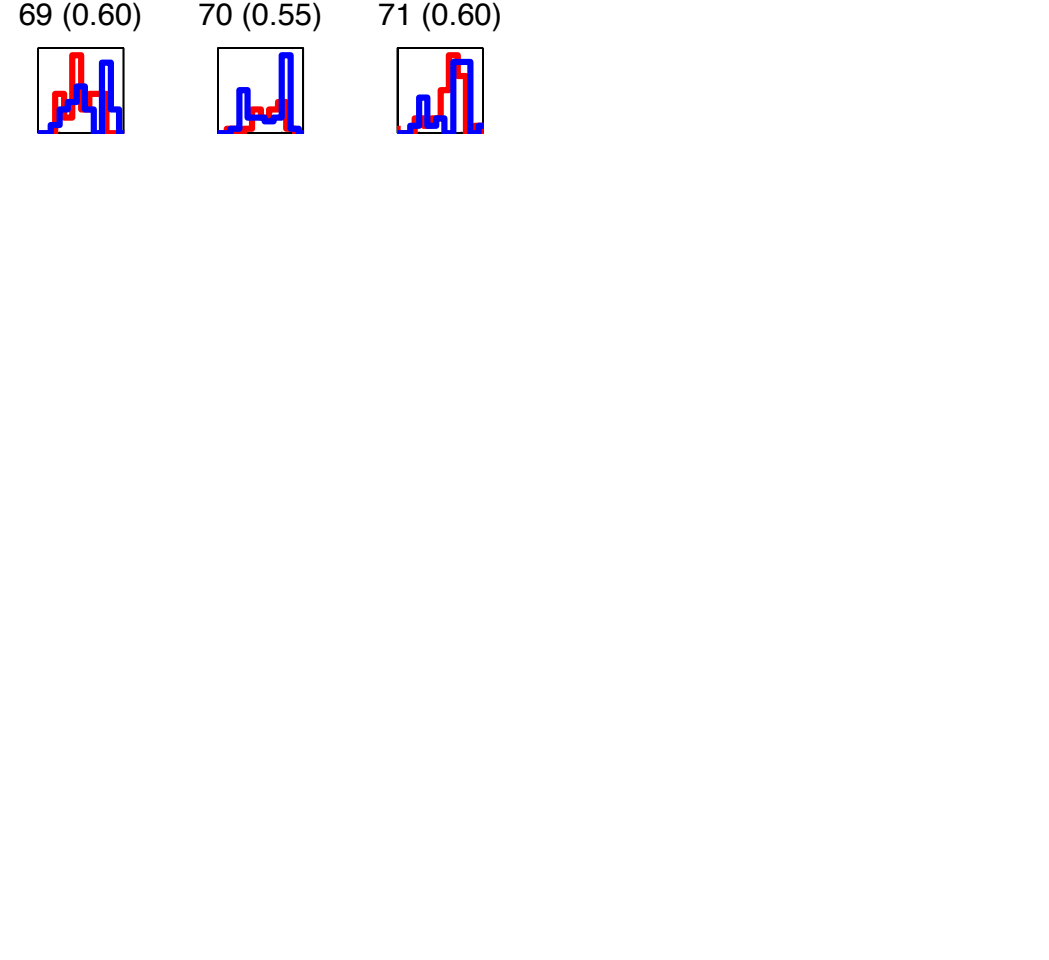

## 4 hr chase

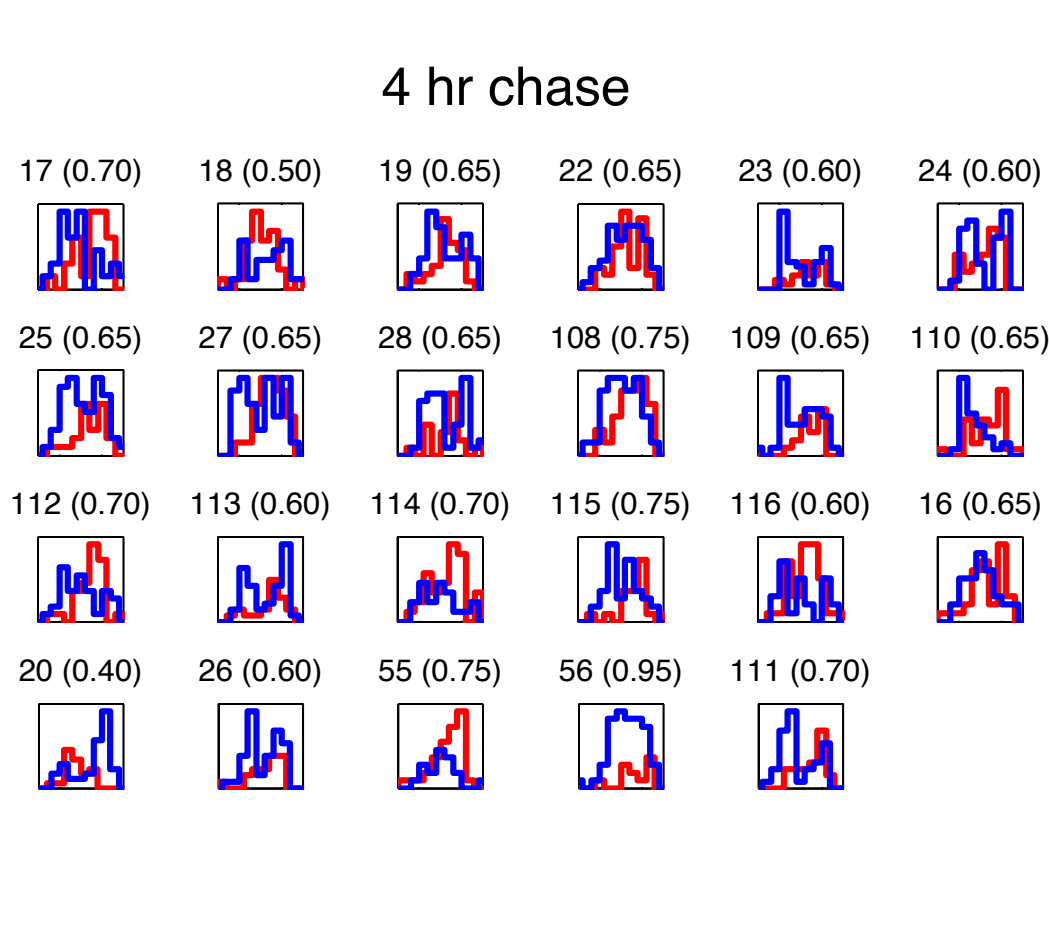

## 5 hr chase

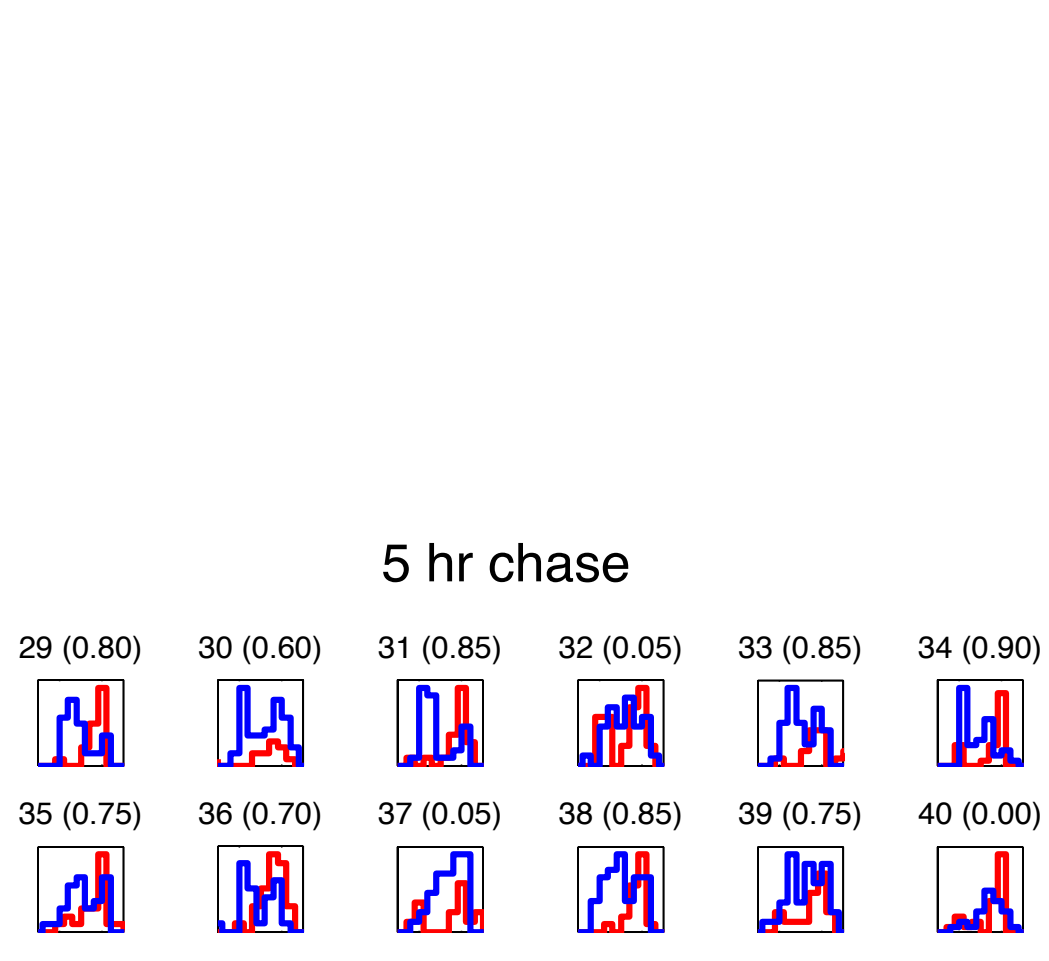

## 6 hr chase

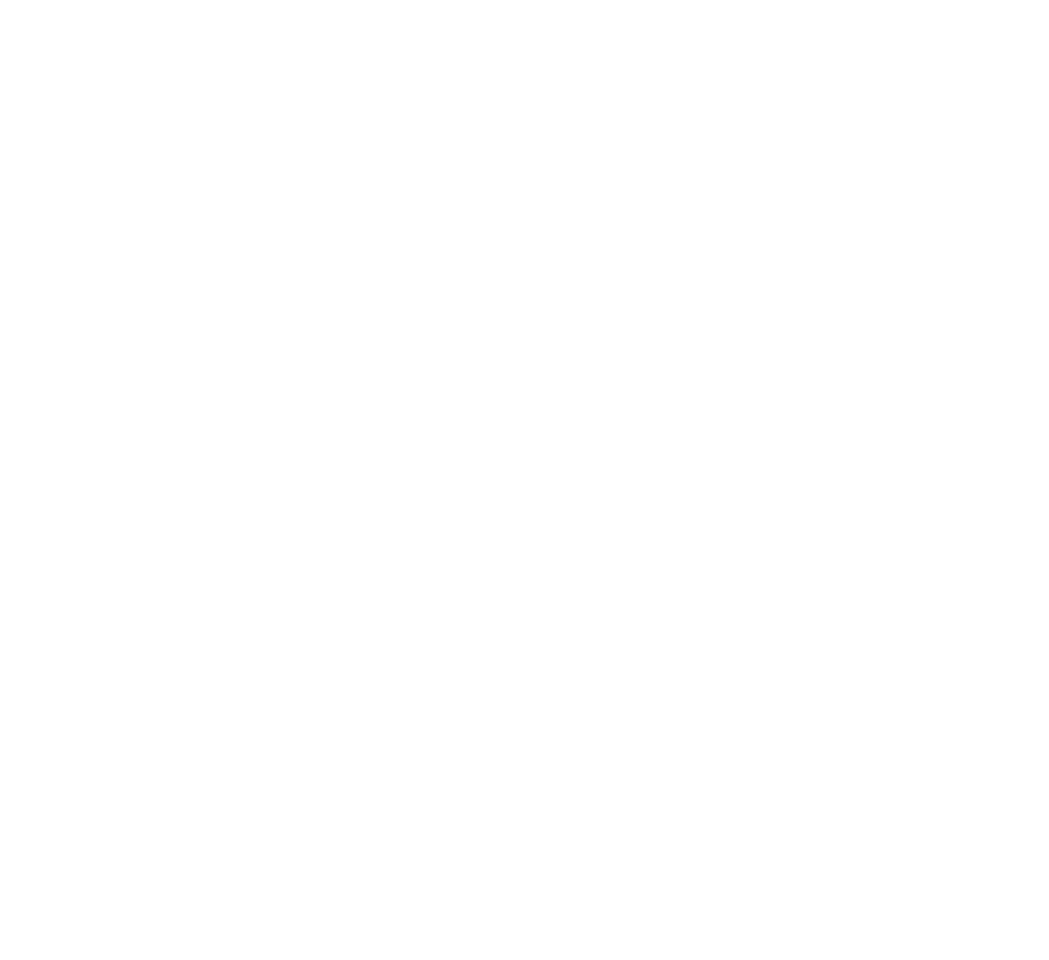

## 8 hr chase

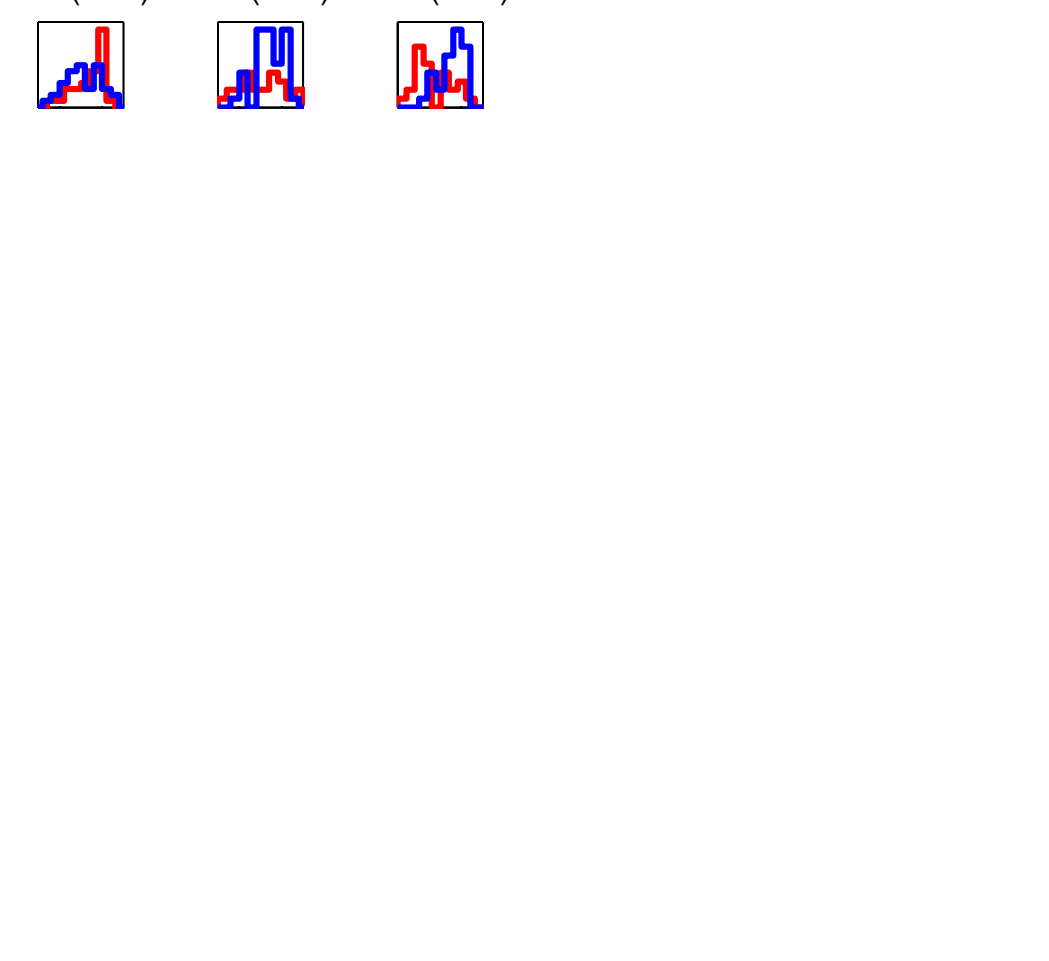

Supplement: S1 Dataset — Numbers displayed on top of histogram are formatted as “gonad_ID (estimated fraction of cell cycle completion)”. (PDF) [file pgen.1005985.s015.pdf]
