# Supplementary material for: Intermittent Stem Cell Cycling Balances Self-Renewal and Senescence of the C. elegans Germ Line
Source: PLoS Genet. 2016 Apr 14;12(4):e1005985. doi: 10.1371/journal.pgen.1005985 (PMC4831802; doi:10.1371/journal.pgen.1005985)
Supplement: S1 Table — Associated with Fig 1. (PDF) [file pgen.1005985.s007.pdf]

Data group	Test	n	Statistic value	p-value	Statistical test
A	Effect of genotype on reproductive capacity		$F_{3,505} = 55$	< 2.3E-16	2-way ANOVA
	Interaction effect		$F_{3,505} = 11$	< 3.1E-7	2-way ANOVA
B	Pairwise differences (except *inx-22; fog-2* vs. *spe-8*)			< 2.1E-4	Tukey's honest significance test
	Pairwise differences for *inx-22; fog-2* vs. *spe-8*			> 0.99	Tukey's honest significance test
C	Brood size from day 7, for females mated at day 0 vs. day 0 + day 5	40 each		> 0.06	Wilcoxon
	Brood size from day 7, for females mated at day 5 vs. day 0 + day 5	40 each		< 0.002	Wilcoxon
