# Supplementary material for: Intermittent Stem Cell Cycling Balances Self-Renewal and Senescence of the C. elegans Germ Line
Source: PLoS Genet. 2016 Apr 14;12(4):e1005985. doi: 10.1371/journal.pgen.1005985 (PMC4831802; doi:10.1371/journal.pgen.1005985)
Supplement: S2 Table — Associated with Fig 2. (PDF) [file pgen.1005985.s008.pdf]

Data group	Sample 1	Average value	n	Sample 2	Average value	n	p-value	Statistical test
A	Mitotic index of fed worms	2.2%	12	Mitotic index of starved worms	0.08%	17	< 0.016	Wilcoxon
B	Number apoptotic cells per gonadal arm of fed worms	18.3	15	Number apoptotic cells per gonadal arm of starved worms	1.9	15	< 2.9E-7	Wilcoxon
C	Brood size fed worms	271	20	Brood size starved worms	527	20	< 8.6E-4	Wilcoxon
D	Number apoptotic cells per gonadal arm of starved worms after recovered on food	15	20	Number apoptotic cells per gonadal arm of control worms	17.1	20	> 0.36	Wilcoxon
