# Supplementary material for: Intermittent Stem Cell Cycling Balances Self-Renewal and Senescence of the C. elegans Germ Line
Source: PLoS Genet. 2016 Apr 14;12(4):e1005985. doi: 10.1371/journal.pgen.1005985 (PMC4831802; doi:10.1371/journal.pgen.1005985)
Supplement: S3 Table — Associated with Fig 3. (PDF) [file pgen.1005985.s009.pdf]

Data group	Sample 1	Average value	n	Sample 2	Average value	n	p-value	Statistical test
A	Ovulation rate *fog-2* on HU	3.8/day	30	Ovulation rate *fog-2* control	3.6/day	30	> 0.74	Wilcoxon
	Ovulation rate *inx-22; fog-2* on HU	24/day	20	Ovulation rate *inx-22; fog-2* control	26/day	20	> 0.79	Wilcoxon
B	Brood size HU-treated *fog-2*	589	47	Brood size control *fog-2*	431	47	< 5.8E-5	Wilcoxon
	Brood size HU-treated *inx-22; fog-2*	398	20	Brood size control *inx-22; fog-2*	199	20	< 0.03	Wilcoxon
C	Lifespan control	19.5 d	96	Lifespan HU-treated	14.3 d	96	< 3.5E-8	Log-rank
	Lifespan at 35°C control	13.8 h	95	Lifespan at 35°C HU-treated	12.7 h	90	< 0.002	Log-rank
D	Incidence of M-phase cells in control	7.1	15	Incidence of M-phase cells after 50 μM CDKI treatment	0.6	15	< 0.0006	Wilcoxon
E	Brood size control	197	40	Brood size following 50 μM CDKI	431	40	< 0.002	Wilcoxon
