# Supplementary material for: Intermittent Stem Cell Cycling Balances Self-Renewal and Senescence of the C. elegans Germ Line
Source: PLoS Genet. 2016 Apr 14;12(4):e1005985. doi: 10.1371/journal.pgen.1005985 (PMC4831802; doi:10.1371/journal.pgen.1005985)
Supplement: S4 Table — Associated with Fig 4. (PDF) [file pgen.1005985.s010.pdf]

Data group	Test	n	p-value	Statistical test used
A	Number of RPA-1::YFP foci/nucleus in day 4 selfed vs. mated	26-28 gonadal arms (20 cells each)	< 0.012	Wilcoxon
	Number of RPA-1::YFP foci/nucleus in selfed day 1 vs. day 4	17-26 gonadal arms (20 cells each)	< 0.0019	Wilcoxon
B	Total brood size *hus-1* vs. wild-type	26 for each	< 0.0003	Wilcoxon
	Brood size days 1-3 *hus-1* vs. wild-type		> 0.9	Wilcoxon
	Brood size day 4-onward hus-1 vs. wild-type		< 2.8E-5	Wilcoxon
