# Supplementary material for: Intermittent Stem Cell Cycling Balances Self-Renewal and Senescence of the C. elegans Germ Line
Source: PLoS Genet. 2016 Apr 14;12(4):e1005985. doi: 10.1371/journal.pgen.1005985 (PMC4831802; doi:10.1371/journal.pgen.1005985)
Supplement: S5 Table — Associated with Fig 6. (PDF) [file pgen.1005985.s011.pdf]

		Genotype, age and mating status							
**Data group**	**Value**	**Wild-type day 1**	**Wild-type day 3**	**Mated wild-type day 3**	***fog-1***	***fog-2***	**Mated *fog-2***	***inx-22; fog-2***	***spe-8***
A	Mitotic index	2.2%	2.0%	2.6%	0.76%	1.4%	2.1%	1.4%	1.8%
B	MI CV 95% CI	0.56 - 0.79	0.72 - 0.89	0.53 - 0.65	1.29 - 1.70	0.96 - 1.25	0.56 - 0.73	0.71 - 0.87	0.52 - 0.69
C	Initial rate of cell cycle progression (cycles / hour)	0.18	0.16	0.19	0.17	0.16	0.18	0.20	0.21
D	Set of chase times considered (hours after pulse)	0,2,3, 4,5,6, 8	0,3,4	0,3,4, 5,6,8	0,2,3	0,2,3	0,2,3, 4,5,6	0,2,3	0,3,6
E	Number of mitotic zone cells	257 ±35	210 ±44	220 ±37	323 ±47	323 ±42	246 ±30	287 ±37	257 ±39
F	Total mitotic zone number in chase times considered	157	107	231	91	47	131	84	96
G	Total mitotic zone number used for MI and cell number	157	214	231	191	146	131	196	96
H	Median EMD distance of best fit	0.19	0.17	0.24	0.24	0.17	0.24	0.24	0.20

S5 Table. Initial rates of cell cycle progression after EdU labeling, quality of underlying fits, and other characteristics of cycling cells.
Rates were computed by considering the largest contiguous set of early chase times for which desynchronization between mitotic zones was minimal (resultant magnitude greater than 0.4), thus minimizing the likelihood of the considered gonads having switched to a dormant state and artificially lowering our estimate. Cell cycle progression was fitted to a linear function of chase time. No pairwise differences are statistically significant ($p > 0.09$ for each pair; p-values computed as described [1]). A subset of used chase times are displayed in Fig. 6C and in S1 Dataset. Experimental DNA

`

content from genotypes and ages that show desynchronization (wild-type day 3, *fog-1*, and *fog-2*) is as well fitted — and in fact very slightly better fitted — by simulations as experimental DNA content from other genotypes and ages (median EMD 0.20 and 0.21, respectively; $p < 0.0076$). This shows that our cell cycle simulation is suitable for the range of experimental data to which it is applied. Also shown are the average mitotic index (MI), the average coefficient of variation (CV) in MI (shown as 95% confidence interval), and the average number of cells found in the mitotic zones of each genotype.

`
