# Supplementary material for: Intermittent Stem Cell Cycling Balances Self-Renewal and Senescence of the C. elegans Germ Line
Source: PLoS Genet. 2016 Apr 14;12(4):e1005985. doi: 10.1371/journal.pgen.1005985 (PMC4831802; doi:10.1371/journal.pgen.1005985)
Supplement: S6 Table — Associated with Fig 7. (PDF) [file pgen.1005985.s012.pdf]

Data group	Measurement	99% BC_a bootstrapped confidence interval
A	Brood size CV when mated at the onset of adulthood	0.077–0.17
B	Brood size CV when mated at day 2 of adulthood	0.21–0.49
C	Brood size CV of brood from day 2 of adulthood onwards, when mated at the onset of adulthood	0.09–0.20
