# Supplementary material for: Intermittent Stem Cell Cycling Balances Self-Renewal and Senescence of the C. elegans Germ Line
Source: PLoS Genet. 2016 Apr 14;12(4):e1005985. doi: 10.1371/journal.pgen.1005985 (PMC4831802; doi:10.1371/journal.pgen.1005985)
Supplement: S7 Table — Associated with Fig 8. (PDF) [file pgen.1005985.s013.pdf]

| Data group | Test | n | p-value | Statistical test used |
|---|---|---|---|---|
| A | Effect of population density on reproductive capacity in *fog-1* at day 4 of adulthood | >26 for each | < 2.1E-7 | Wilcoxon |
| B | Effect of population density on mitotic zone dormancy in *fog-1* at day 1 of adulthood | 71 grouped; 69 singled | < 0.046 | Fisher's exact test |
| C | Effect of population density on mitotic zone dormancy in N2 at day 3 of adulthood | 103 grouped; 64 singled | < 0.006 | Fisher's exact test |
| D | Effect of population density on mitotic zone dormancy in *daf-22* at day 3 of adulthood | 90 grouped; 72 singled | > 0.52 | Fisher's exact test |
