# Supplementary material for: Intermittent Stem Cell Cycling Balances Self-Renewal and Senescence of the C. elegans Germ Line
Source: PLoS Genet. 2016 Apr 14;12(4):e1005985. doi: 10.1371/journal.pgen.1005985 (PMC4831802; doi:10.1371/journal.pgen.1005985)
Supplement: S8 Table — Associated with Fig 9. (PDF) [file pgen.1005985.s014.pdf]

Data group	Sample 1	Average value	n	Sample 2	Average value	n	p-value	Statistical test
A	number of diplotene cells time point 1: *fog-1*	32	10	number of diplotene cells time point 2: *fog-1*	21	10	< 0.016	Wilcoxon
	number of diplotene cells time point 2: *fog-1*	21	10	number of diplotene cells time point 4: *fog-1*	39	10	< 0.003	Wilcoxon
	number of diakinesis cells time point 1: *fog-1*	19	10	number of diakinesis cells time point 2: *fog-1*	10	10	< 2E-4	Wilcoxon
	number of diakinesis cells time point 2: *fog-1*	10	10	number of diakinesis cells time point 4: *fog-1*	15	10	< 0.016	Wilcoxon
	number of diplotene cells time point 1: *inx-22; fog-2*	25	10	number of diplotene cells time point 2: *inx-22; fog-2*	31	10	> 0.07	Wilcoxon
	number of diplotene cells time point 2: *inx-22; fog-2*	31	10	number of diplotene cells time point 4: *inx-22; fog-2*	30	10	> 0.7	Wilcoxon
	number of diakinesis cells time point 1: *inx-22; fog-2*	10	10	number of diakinesis cells time point 2: *inx-22; fog-2*	14	10	< 0.016	Wilcoxon
	number of diakinesis cells time point 2: *inx-22; fog-2*	14	10	number of diakinesis cells time point 4: *inx-22; fog-2*	16	10	> 0.23	Wilcoxon
B	*fog-2* number of progeny	50	30	*ced-3; fog-2* number of progeny	48	30	> 0.5	Wilcoxon
C	0 h after mating	17.1	20	8 h after mating	9.2	15	< 0.003	Wilcoxon
