# Supplementary material for: Intermittent Stem Cell Cycling Balances Self-Renewal and Senescence of the C. elegans Germ Line
Source: PLoS Genet. 2016 Apr 14;12(4):e1005985. doi: 10.1371/journal.pgen.1005985 (PMC4831802; doi:10.1371/journal.pgen.1005985)
Supplement: S1 Text — (PDF) [file pgen.1005985.s017.pdf]

**Supporting Text**: Cell cycle analysis and computational simulations

1. Cell cycle analysis

Retrieving single-organ cell cycle data is important given stochastic switching between active and dormant states, which could be obscured in population-level studies that aggregate data across organs. A number of methods of assaying the *C. elegans* germline cell cycle have already been reported [2-4]. These methods rely on labeling cells in S phase and measuring the time it takes for labeled or unlabeled cells to reach another phase for which convenient markers exist (for example, S or M phase). Information about phases that were not assayed with a specific marker is missing, as is information about progression through S phase: S phase markers do not trivially distinguish between cells at the beginning or at the end of S phase, which are hours apart. A novel aspect of our technique is that it makes extensive use of the total cell DNA content information, which is retrieved from three-dimensional segmentations of confocal images. This gives a more complete picture of cell cycle progression after pulse labeling, making it possible to assay cell cycle progression of single gonadal arms, and thus avoiding the need to pool data and to rely on population averages.

Our cell cycle analysis relies on cell-by-cell quantification of EdU and DNA contents. We thus developed software to segment individual cells in three-dimensional images of mitotic zones acquired by confocal microscopy (image segmentation is the process of partitioning image pixels into distinct subsets; [5]). Our approach to germ cell segmentation can be divided into three steps: automated detection of cell centers, manual curation of detected centers, and running of active contours [6] initialized from curated cell centers (see S5 Figure A—B for example segmentation results, and also [5,7]). We quantified DNA and EdU content from masks generated by our segmentation pipeline, considering only cells at the top of the stacks to avoid attenuation of fluorescence signal intensity due to tissue absorption or scattering, and focusing on the distal-most 12 rows since they contain the majority of the ~130-160 mitotic zone cells that are actively cycling [4], and do not contain a substantial portion of premeiotic S phase cells that could confound the cell cycle analysis. The region analyzed was reduced to 10 rows for wild-type hermaphrodites at day 3 of adulthood, to account for shortening of the mitotic zone.

As outlined above, pulse chase experiments assaying the cell cycle rely on pulse-labeling cells that are in a specific phase, and measuring cell cycle progression of labeled and unlabeled cells as chase time increases. We used EdU to mark cells that were in S phase at the time of the pulse, and total cell DNA content at the end of the chase to measure cell cycle progression. Although in theory DNA content distinguishes between the phases of the cell cycle (with the exception of G2/M, which both have maximal DNA content), in practice limitations in measurement precision make it difficult to find appropriate thresholds to distinguish between G1 and S, and between S and G2/M. Therefore, instead of trying to identify cell cycle phase based just on DNA content, we computed DNA content histograms of EdU-positive and EdU-negative cells after the chase. Cells were determined as EdU-positive or -negative by thresholding their quantified EdU content [5]. We then fitted these experimental histograms to histograms

derived from simulations of germ cell cycling that proceeded for increasing amounts of time, and asked what simulated time — expressed as a fraction of total cell cycle length — gave the best fit to experimental data.

The simulations used to fit experimental data made the following assumptions: (1) cells in the mitotic zone had uniform cell cycle lengths, (2) dividing cells pushed out neighbors, causing a chain of displacements eventually resulting in a cell being evicted from the mitotic zone, and (3) the initial cellular age distribution was exponential. A series of simulated DNA content histograms were generated for EdU-positive and EdU-negative cell populations across a series of 20 chase times after an EdU pulse (S5 Figure D; S1 Movie). For each pair of experimental EdU-positive and EdU-negative histograms of DNA contents generated on a gonad-by-gonad basis, the pair of simulated histograms that best matched the experimental histograms was identified to estimate the cycle "phase" of the mitotic zone as a whole (S5 Figure E; see S1 Dataset for a large set of experimental histograms marked with best fit). Inter-histogram distances were computed using the circular Earth Mover's Distance (EMD), customized to work on periodic values [8] (see [7] for more detail). It is necessary to take periodicity into account because a cell that is about to divide and a cell that has just divided have a two-fold difference in DNA content and are at the extremes of the DNA content axis, but are very close in cell cycle phase; for our purposes, minimal and maximal contents should thus be considered the same (see S5 Figure F for histograms that are close together in cell cycle time but far apart in DNA content). Analysis of wild-type hermaphrodites at the first day of adulthood using our new technique gave results — an overall cell cycle length of 5.5 hr — that are close to those established by others [3,4] and by us using alternative methods [7]. For displaying purposes in Figure 6C, the average phase at chase time 0 was defined as the origin for each genotype and subtracted from the phases computed for subsequent chase times; jitter was added to the plots in Figure 6C (produced using CircStat [9]) to allow visualization of squares that would otherwise perfectly overlap.

We asked whether fits to our simple model were adversely affected by gonads of different genotypes and ages differing in their cycle properties in respects other than the frequency of active/dormant state switching. To this end, we computed the average distances of experimental DNA content histograms to the best simulated fit for the range of genotypes and ages that we studied. We found that young adult wild-type — for which our results agree with other reports — did not give a better overall fit than any of the of other datasets we report here (S5 Table). Although we do not exclude that there could be more subtle changes of cell cycle in the mitotic zone than we report here, we conclude that the technique we report here is suitable to assay overall gonad cell cycle activity.

2. Simulation of stochastic reproductive potential capacity decrease with replicative age

We coded a simple, agent-based simulation with gonadal arms that switched stochastically between active and dormant states. In the active state, gonadal arms lost reproductive capacity at a steady rate. In the dormant state, no reproductive capacity was lost. The amount of time spent in each state followed a log-normal distribution with location and shape parameters set to 2, to reproduce the reproductive capacity CV of ~0.3 measured at day 2 (interesting questions for future studies will be to determine whether the switching process is stationary, and to more finely characterize the experimental distribution of the waiting times between switches). The rate at which reproductive capacity is lost in the active state was set to 9.4 progeny / h, such that, starting from an initial reproductive capacity of 846 (derived from *fog-1* data), after two days of intermittent cycling that capacity has dropped to ~620 (also derived from *fog-1* experimental data). Initial reproductive capacities were draw from a normal distribution with CV 0.12 (as measured experimentally). With the parameters chosen, the CV in remaining reproductive capacity was 0.31, close to the experimentally-measured value. Code to reproduce these results is available at https://github.com/cinquin/StoCySim

**Supporting material references**


1. Clogg CC, Petkova E, Haritou A (1995) Statistical methods for comparing regression coefficients between models. American Journal of Sociology: 1261–1293.

2. Crittenden SL, Leonhard KA, Byrd DT, Kimble J (2006) Cellular analyses of the mitotic region in the Caenorhabditis elegans adult germ line. Mol Biol Cell 17: 3051–3061. doi:10.1091/mbc.E06-03-0170.

3. Jaramillo-Lambert A, Ellefson M, Villeneuve AM, Engebrecht J (2007) Differential timing of S phases, X chromosome replication, and meiotic prophase in the C. elegans germ line. Dev Biol 308: 206–221. doi:10.1016/j.ydbio.2007.05.019.

4. Fox PM, Vought VE, Hanazawa M, Lee M-H, Maine EM, et al. (2011) Cyclin E and CDK-2 regulate proliferative cell fate and cell cycle progression in the C. elegans germline. Development 138: 2223–2234. doi:10.1242/dev.059535.

5. Chiang M, Hallman S, Cinquin A, de Mochel NR, Paz A, et al. (2015) Analysis of in vivo single cell behavior by high throughput, human-in-the-loop segmentation of three-dimensional images. BMC Bioinformatics 16: 397. doi:10.1186/s12859-015-0814-7.

6. Ortiz de Solorzano C, Malladi R, Lelièvre SA, Lockett SJ (2001) Segmentation of nuclei and cells using membrane related protein markers. J Microsc 201: 404–415.

7. Chiang M, Cinquin A, Paz A, Meeds E, Price CA, et al. (2015) Control of C. elegans germline stem cell cycling speed meets requirements of design to minimize mutation accumulation. BMC Biol 13: 51. doi:10.1186/s12915-015-0148-y.

8. Rabin J, Delon J, Gousseau Y (2008) Circular Earth Mover's Distance for the comparison of local features pp. 1–4.

9. Berens P (2009) CircStat: A MATLAB Toolbox for Circular Statistics. Journal of Statistical Software 31: 1–21.
